# Nasopharyngeal competition dynamics are likely to be altered following vaccine introduction: bacteriocin prevalence and diversity among Icelandic and Kenyan pneumococci

**DOI:** 10.1099/mgen.0.001060

**Published:** 2023-07-12

**Authors:** Madeleine E. B. Butler, Melissa J. Jansen van Rensburg, Angela Karani, Benedict Mvera, Donald Akech, Asma Akter, Calum Forrest, Andries J. van Tonder, Sigríður J. Quirk, Gunnsteinn Haraldsson, Stephen D. Bentley, Helga Erlendsdóttir, Ásgeir Haraldsson, Karl G. Kristinsson, J. Anthony G. Scott, Angela B. Brueggemann

**Affiliations:** ^1^​ Imperial College London, London, UK; ^2^​ University of Oxford, Oxford, UK; ^3^​ KEMRI Wellcome Trust Programme, Kilifi, Kenya; ^4^​ University of Cambridge, Cambridge, UK; ^5^​ University of Iceland and Landspitali - The National University Hospital of Iceland, Reykjavík, Iceland; ^6^​ Wellcome Sanger Institute, Hinxton, UK; ^7^​ University of Iceland and Children’s Hospital Iceland, Landspitali, Reykjavík, Iceland; ^8^​ London School of Hygiene and Tropical Medicine, London, UK

**Keywords:** bacteriocins, competition, pneumococcus, vaccine-mediated changes

## Abstract

Bacteriocins are antimicrobial peptides produced by bacteria to inhibit other bacteria in the surrounding environment. *Streptococcus pneumoniae* is a leading cause of disease worldwide and colonises the healthy human nasopharynx, where it competes for space and nutrients. Pneumococcal conjugate vaccines have reduced the incidence of disease, but they also restructure the bacterial population, and this restructuring likely alters the nasopharyngeal competition dynamics. Here, the distribution of bacteriocins was examined in over 5000 carriage and disease-causing pneumococci from Iceland and Kenya, recovered before and after the introduction of pneumococcal vaccination. Overall, up to eleven different bacteriocin gene clusters were identified per pneumococcus. Significant differences in the prevalence of bacteriocins were observed before and after vaccine introduction, and among carriage and disease-causing pneumococci, which were largely explained by the bacterial population structure. Genetically similar pneumococci generally harboured the same bacteriocins although sometimes different repertoires of bacteriocins were observed, which suggested that horizontal transfer of bacteriocin clusters had occurred. These findings demonstrated that vaccine-mediated changes in the pneumococcal population altered the prevalence and distribution of bacteriocins. The consequences of this for pneumococcal colonisation and disease remain to be determined.

## Data Summary

Analysis code is available at GitHub: https://github.com/brueggemann-lab/bacteriocins_IceKen_2022. Assembled genomes are publicly available from PubMLST (https://pubmlst.org/organisms/streptococcus-pneumoniae; Tables S2 and S3). All Supplementary Materials may be downloaded from Figshare: https://doi.org/10.6084/m9.figshare.23266526.v1[1].

**Impact Statement**

Pneumococcal conjugate vaccines (PCVs) are one of the most influential public health successes over the past two decades and PCV implementation has significantly reduced morbidity and mortality worldwide. However, one consequence of PCV-induced immunity is that the dynamics of nasopharyngeal colonisation among healthy individuals is altered and since colonisation precedes disease it is important to understand these changes. Bacteriocins are believed to be involved in competition between bacteria colonising the same ecological niche. Here, our work has shown that each pneumococcal genome possessed a repertoire of bacteriocin clusters and that the prevalence of pneumococcal bacteriocins pre- and post-PCV implementation changed. This was largely because the pneumococci circulating post-PCV were different, and these changes have relevance for both colonisation and disease. Pneumococcal bacteriocins are complicated in terms of their high prevalence and distribution among pneumococci, but there are also clear patterns emerging from this work that provide the critical framework for understanding bacteriocins and their role in pneumococcal biology and competition.

## Introduction

*Streptococcus pneumoniae* (pneumococcus), is a major cause of lower respiratory tract infections (LRTI), invasive pneumococcal disease (IPD) including meningitis and bacteraemia, and otitis media (OM). Despite vaccines and antimicrobials, in 2016 it was estimated that pneumococcus caused 197 million episodes of pneumonia and more than 1.1 million deaths worldwide, disproportionately affecting low- and middle-income countries [2].

The ecological niche of the pneumococcus is the paediatric nasopharynx and whilst carriage is usually asymptomatic, it is a precursor to disease [3]. Carriage duration varies between serotypes, and less invasive serotypes typically colonise for longer periods of time [4]. Since pneumococci and other bacterial species in the nasopharynx are competing for space and resources, competition dynamics are likely to be associated with pneumococcal pathogenesis, although the mechanism of competition is not yet understood [5, 6]. Furthermore, pneumococcal conjugate vaccines (PCVs) target a limited number of serotypes, and after PCV implementation vaccine serotypes typically decrease and nonvaccine serotypes increase within the human population, which results in changes to nasopharyngeal competition dynamics [7–9].

Bacteria can compete with bacteriocins, which are peptides or small proteins that give the producing bacteria an advantage by killing or inhibiting other bacteria in the environment [10–12]. Bacteriocins are encoded on biosynthetic gene clusters, which in the simplest form encode a toxin plus an ‘immunity’ gene(s) to protect the producing strain from its own bacteriocin. Bacteriocin clusters may also encode genes for post-translational modification enzymes, dedicated transporters, or regulatory systems [12]. The most well-studied pneumococcal bacteriocin is the bacteriocin-like peptide (*blp*) cluster, which is highly diverse and ubiquitous [10, 13, 14]. More recently, genome mining studies revealed a wide repertoire of different bacteriocin clusters among large and diverse pneumococcal datasets; however, the prevalence and diversity of bacteriocins within well-defined subsets of the pneumococcal population has not been investigated [13, 15, 16].

Therefore, the aim of this study was to investigate bacteriocin clusters among pneumococci recovered from disease and carriage in Iceland and Kenya, before and after the introduction of 10-valent PCV (PCV10) immunisation. In total, over 5000 pneumococcal genomes were screened for 20 different bacteriocin clusters, which revealed that bacteriocins were widespread, were associated with the underlying pneumococcal population structure in each country, and that the prevalence and distribution of these bacteriocins were significantly altered post-PCV10. Overall, these findings suggest that nasopharyngeal competition dynamics are likely to be altered following PCV introduction.

## Methods

### Pneumococcal isolate collections, microbiology, and serotyping

Icelandic carriage pneumococci were recovered from nasopharyngeal swabs of healthy children <7 years of age in day care centres in the Reykjavik area. Swabs were collected in March every year from 2009 to 2014, except in 2009 when 40 % of the samples were taken in April. Disease pneumococci were recovered from diagnostic specimens sent to the clinical microbiology laboratory at Landspitali University Hospital from 2009 to 2014; OM isolates were collected from the middle ear of children <7 years of age with OM; LRTI isolates were collected from the sputum of adults >18 years of age with suspected pneumonia; and invasive pneumococci were collected from normally sterile sites of patients of all ages with IPD. All of the Icelandic IPD isolates collected between 2009 and 2014, and every other carriage, LRTI and OM isolate in the overall dataset were selected for whole genome sequencing. The pneumococci recovered from carriage, LRTI and OM were published previously [8, 9].

Kenyan isolates were selected for whole genome sequencing from pneumococcal collections at the KEMRI-Wellcome Trust Research Programme, and were recovered from residents of the Kilifi Health and Demographic Surveillance System (KHDSS) [7]. Carriage pneumococci were recovered from healthy patients of all ages in the KHDSS who had been recruited to carriage studies in the district [7, 17–20]. Isolates collected between 2004 and 2017, excluding 2011 (the year PCV10 was introduced), were sampled randomly from the age groups represented by the carriage studies: 3–5, 6–11, 12–23 and 24–50 months, 5–14, 15–64 and 65+ years. Sampling within each age stratum was weighted to reflect the observed population structure of residents of the KHDSS. Invasive pneumococci were collected from patients of all ages presenting with IPD at Kilifi County Hospital, and all isolates collected from 2003 to 2017 were included in this study.

All pneumococci were cultured from primary specimens and identified using standard microbiological techniques. Isolates were serotyped in Kenya using latex agglutination and confirmed with the Quellung reaction if necessary. Pneumococci in Iceland were serotyped using latex agglutination and confirmed with multiplex PCR assays and sequence-based serotyping when needed [8, 9, 21, 22].

### DNA extraction and whole genome sequencing

Freezer stocks of pneumococcal isolates were cultured using standard microbiological methods. Optochin disc susceptibility confirmed that isolates were pneumococci and any viridans streptococci were excluded from further processing. DNA was extracted as described previously [8] and sequenced using the Illumina HiSeq2000 platform.

### Genome assembly, quality control, MLST and clonal complex definitions

Draft genomes were assembled *de novo* into contigs from short paired-end reads using an internal Sanger pipeline that utilised Velvet Optimiser, SSPACE and GapFiller [23–25]. Isolate records with corresponding provenance data and assembled genomes were stored in a private PubMLST database for analysis during this study [26]. Sequence assembly statistics (i.e. genome size, number of contigs, GC content) were inspected and any genome with a value greater than two standard deviations from the mean of the dataset for any of the assembly statistics was investigated manually.

The ribosomal multilocus sequence typing (rMLST) species identification tool (https://pubmlst.org/species-id) was used to confirm that genomes were pneumococcal sequences [27]. Within PubMLST, genomes were automatically screened and assigned multilocus sequence typing (MLST) and rMLST alleles, and any new alleles and sequence types (STs) were submitted to PubMLST curators for assignment (https://pubmlst.org/organisms/streptococcus-pneumoniae). MLST and rMLST data were investigated manually to identify any locus with >1 allele assignment, which suggested contamination. Suboptimal genomes were excluded from further analyses.

Phyloviz was used to define clonal complexes (CCs) based upon founder STs and closely related single locus variants (SLVs), using the entire PubMLST database of unique STs. CCs were named after the founder ST [28]. A pneumococcus designated a ‘singleton’ did not have any SLVs that connected it to any other ST.

### *In silico* serotyping

Icelandic pneumococci were previously serotyped *in silico* with the seqSerotyper tool [8, 21] and SeroBA was used to predict the serotypes of the Kenyan genomes [29]. Discrepancies between phenotypic and *in silico* serotypes in the Kenyan dataset were resolved manually by comparisons to reference capsular polysaccharide (*cps*) locus sequences, but if this was unclear then the phenotypic serotype was used in analyses.

### Annotation of bacteriocin coding sequences

Previously described bacteriocin coding sequences (hereafter simply referred to as ‘genes’) were defined within PubMLST and then study genomes were automatically screened for these genes using blast searches (using thresholds of >70 % sequence identity and >50 % alignment length) [15, 26]. Alleles were assigned when matches to existing alleles were identified, and any putatively new alleles were manually curated by visual inspection. Alleles were assigned to full length bacteriocin gene sequences and those with insertions and deletions. A bacteriocin gene was considered to be absent if there was no match to an existing allele at >50 % sequence identity and at least 30 % gene length.

Streptolancidins A and J each contained a pair of small genes that were indistinguishable at a sequence level (*slaA1* and *slaA2*, *sljA1* and *sljA2*), and two genes from streptolancidin E (*sleT* and *sleX1*) were typically found as a small fragment elsewhere in the pneumococcal genome. The location of each set of genes was determined using the *in silico* hybridisation tool within PubMLST, and each gene was annotated based on its location within the genome. The streptolancidin E genes were annotated only when they occurred as part of a full streptolancidin E cluster.

### Validation of bacteriocin biosynthetic gene clusters

Full and partial bacteriocin biosynthetic gene clusters (hereafter ‘bacteriocin clusters’) were pooled together for subsequent analyses: full clusters possessed the expected set of genes for that cluster (Table S1, available in the online version of this article); and partial clusters were incomplete but more than half of the expected genes were present. Partial clusters were included in analyses because previous experimental work demonstrated that genes in partial bacteriocin clusters were expressed [15]. Single bacteriocin cluster genes and bacteriocin cluster fragments that contained fewer than half of the expected genes were excluded from further analyses.

Because the bacteriocin alleles were assigned gene-by-gene, it was necessary to ensure that the bacteriocin genes were assembled as contiguous clusters. This was assessed computationally and a bacteriocin cluster was deemed contiguous if there were no gaps >2.5 Kb between each of the constituent bacteriocin cluster genes (Figure S1). Bacteriocin clusters that did not meet these criteria were excluded from further analyses. Significant differences in bacteriocin cluster prevalence were determined using the Chi-square test. Figures were generated using Affinity Designer v.1.10.1 (https://affinity.serif.com/en-gb/designer/).

## Results

### Genomic datasets for bacteriocin analyses

Overall, 5071 pneumococcal genomes were analysed for bacteriocin clusters. Of these, 1912 Icelandic pneumococcal genomes were included: 1733 carriage and non-invasive disease pneumococci that were previously published (although four were lower quality genome assemblies and excluded from these bacteriocin analyses; Table S2) [8, 9, 21, 22]; plus 183 new genomes of pneumococci recovered from patients with invasive disease and collected over the same time period (Table 1). In total, 3159 Kenyan genomes were analysed: 3372 pneumococci were selected for inclusion in this study; 3258 genomes were sequenced; and 99 genomes were excluded following quality control assessment (mainly duplicate genomes; Table S3).

**Table 1.** Pneumococcal genomes in the Icelandic and Kenyan study datasets

		No. of isolates recovered from:	
		Iceland	Kenya
Total		1912	3159
Carriage		983	2387
All disease		929	772
	IPD	183	772
	LRTI	283	0
	OM	463	0
Pre-PCV10		1039	1660
Post-PCV10		873	1499

Note: IPD, invasive pneumococcal disease; LRTI, lower respiratory tract infection; OM, otitis media; PCV10, 10-valent pneumococcal conjugate vaccine.

**Fig. 1.**
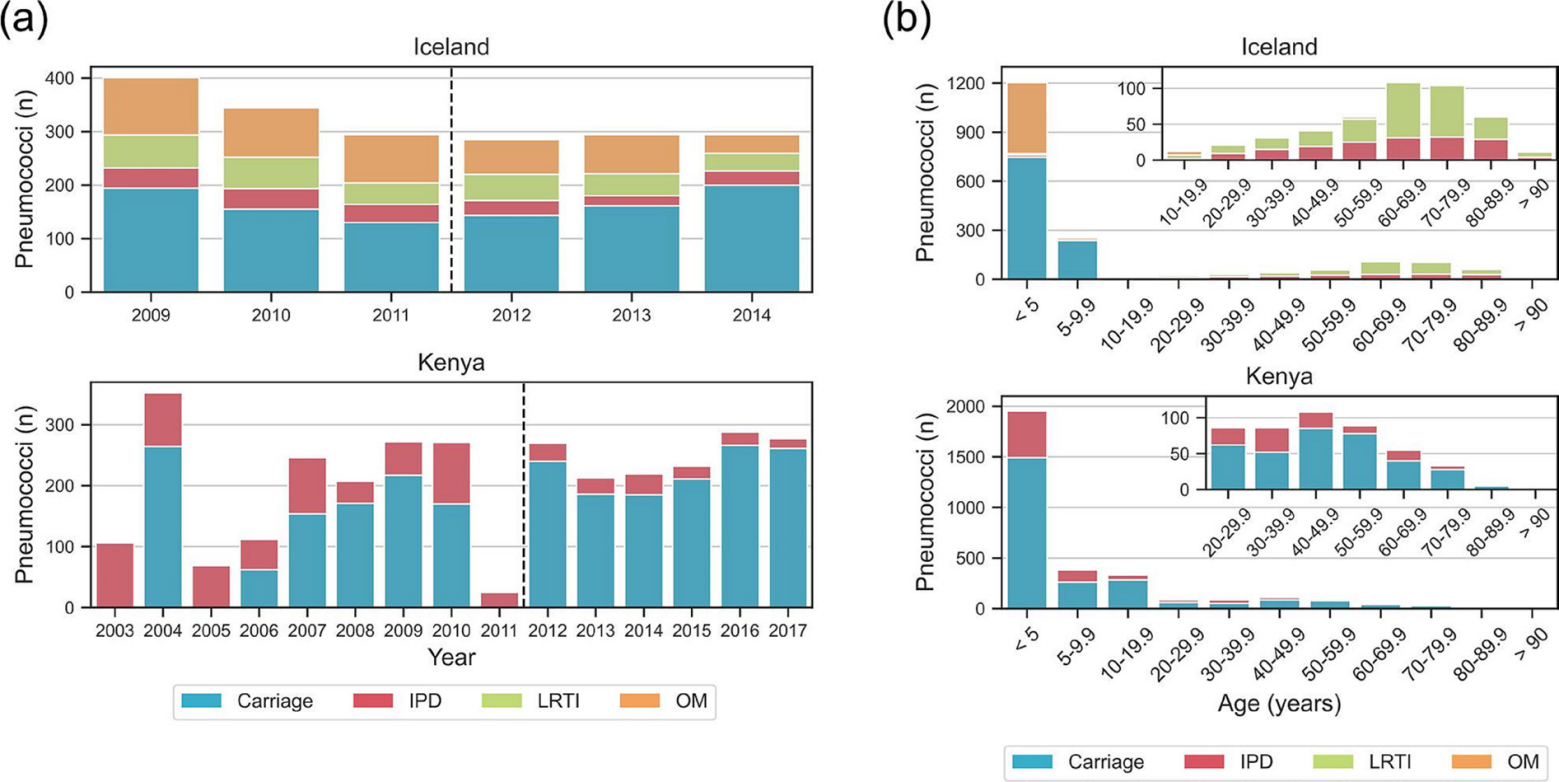
Description of the Icelandic and Kenyan datasets. (**a**) Numbers of carriage and disease pneumococci collected by year of isolation. The dashed line separates pre- and post-PCV10 periods. (**b**) Number of pneumococci collected by age group of study subjects. Inset plots increase data resolution for the older age groups. Note: IPD, invasive pneumococcal disease; LRTI, lower respiratory tract infection; OM, otitis media.

Pneumococci were collected over 6 years (2009–2014) in Iceland and 14 years (2003–2017) in Kenya (Fig. 1a). PCV10 was included in national infant immunisation programmes from 2011 onwards in both countries; therefore, the pre-vaccination period was the years up to and including 2011, and the post-vaccination period was from 2012 onwards. Disease-causing pneumococci were recovered from patients of all ages in both countries. Carriage sampling regimes differed in the two locations: pneumococci were recovered from children <7 years of age and attending day care centres in Iceland (*n*=983); and from participants of all ages in Kenya (total *n*=2387, of which 1587 were recovered from children <7 years of age; Fig. 1b).

### Icelandic and Kenyan pneumococci represent distinct populations

Fifty-nine unique CCs (and five singletons) were observed in the Icelandic dataset, and 116 unique CCs (and 62 singletons) were observed in the Kenyan dataset (Table 2). There were 18 CCs that were represented in both datasets. Overall, 40 and 56 serotypes were observed in the Icelandic and Kenyan datasets, respectively. The most prevalent serotype in both countries was 19F, which was dominant in the Icelandic dataset due to the incidence of OM caused by CC236/271/320 pneumococci in Iceland during the study period [8]. Other serotypes differed between the two populations, e.g. the highly invasive serotype one was nearly equal in prevalence to serotype 19F in the Kenyan dataset but serotype one was rare in the Icelandic dataset (Table 3).

**Table 2.** The 20 most prevalent clonal complexes (CCs) in the Icelandic and Kenyan datasets

Iceland		Kenya	
CC	n (%)	CC	n (%)
236/271/320	293 (15.3)	5902	239 (7.6)
439	217 (11.3)	217	223 (7.1)
199	179 (9.4)	701	163 (5.2)
138/176	122 (6.4)	5339	142 (4.5)
180	107 (5.6)	1146	139 (4.4)
62	94 (4.9)	138/176	133 (4.2)
97	87 (4.6)	156/162	131 (4.1)
490	74 (3.9)	991	104 (3.3)
124	62 (3.2)	230	92 (2.9)
433	61 (3.2)	852	78 (2.5)
30	60 (3.1)	5258	77 (2.4)
392	47 (2.5)	63	70 (2.2)
156/162	46 (2.4)	289	69 (2.2)
344	37 (1.9)	347	62 (2.0)
15	36 (1.9)	914	61 (1.9)
1262	35 (1.8)	7053	58 (1.8)
448	29 (1.5)	702	58 (1.8)
193	28 (1.5)	854	57 (1.8)
100	25 (1.3)	499	55 (1.7)
90	22 (1.2)	1381	49 (1.6)
Other CCs^∗^	235 (12.3)	Other CCs^∗^	954 (30.2)
Singletons	16 (0.8)	Singletons	145 (4.6)

*‘Other CCs’ represent 44 CCs in Iceland and 158 CCs in Kenya.

**Table 3.** The 20 most prevalent serotypes in the Icelandic and Kenyan datasets

Iceland		Kenya	
Serotype	n (%)	Serotype	n (%)
19F*	331 (17.3)	19F*	228 (7.2)
23F*	180 (9.4)	1*	224 (7.1)
6A	163 (8.5)	6A	206 (6.5)
19A	145 (7.6)	19A	153 (4.8)
6B*	122 (6.4)	15BC	146 (4.6)
3	109 (5.7)	35B	139 (4.4)
11A	93 (4.9)	15A	136 (4.3)
15BC	93 (4.9)	14*	131 (4.1)
14*	90 (4.7)	6E(6Bii)	131 (4.1)
nontypable	70 (3.7)	23F*	119 (3.8)
22F	61 (3.2)	11A	117 (3.7)
23A	51 (2.7)	13	98 (3.1)
23B	47 (2.5)	16F	96 (3.0)
35B	33 (1.7)	23B	90 (2.8)
9V*	32 (1.7)	3	90 (2.8)
21	29 (1.5)	34	89 (2.8)
6C	29 (1.5)	10A	82 (2.6)
33F	29 (1.5)	5*	69 (2.2)
6E(6Bii)	26 (1.4)	9V*	63 (2.0)
16F	26 (1.4)	21	62 (2.0)
Other serotypes†	153 (8.0)	Other serotypes	690 (21.8)

*PCV10 serotypes are indicated in bold font.

†‘Other serotypes’ represents additional serotypes in Iceland (*n*=20) and Kenya (*n*=36).

### Bacteriocin clusters were widely distributed among Icelandic and Kenyan pneumococci

In total, the presence or absence of 116 genes associated with 20 putative bacteriocin clusters was investigated among 5071 pneumococcal genomes (Table S3) [13, 15, 30–33]. Partial clusters were consistently observed for streptococcins B and E, and streptolancidins B, C, E and J (Table S4). We found that 95–100 % of bacteriocin genes were in contiguous clusters (Table S5).

**Fig. 2.**
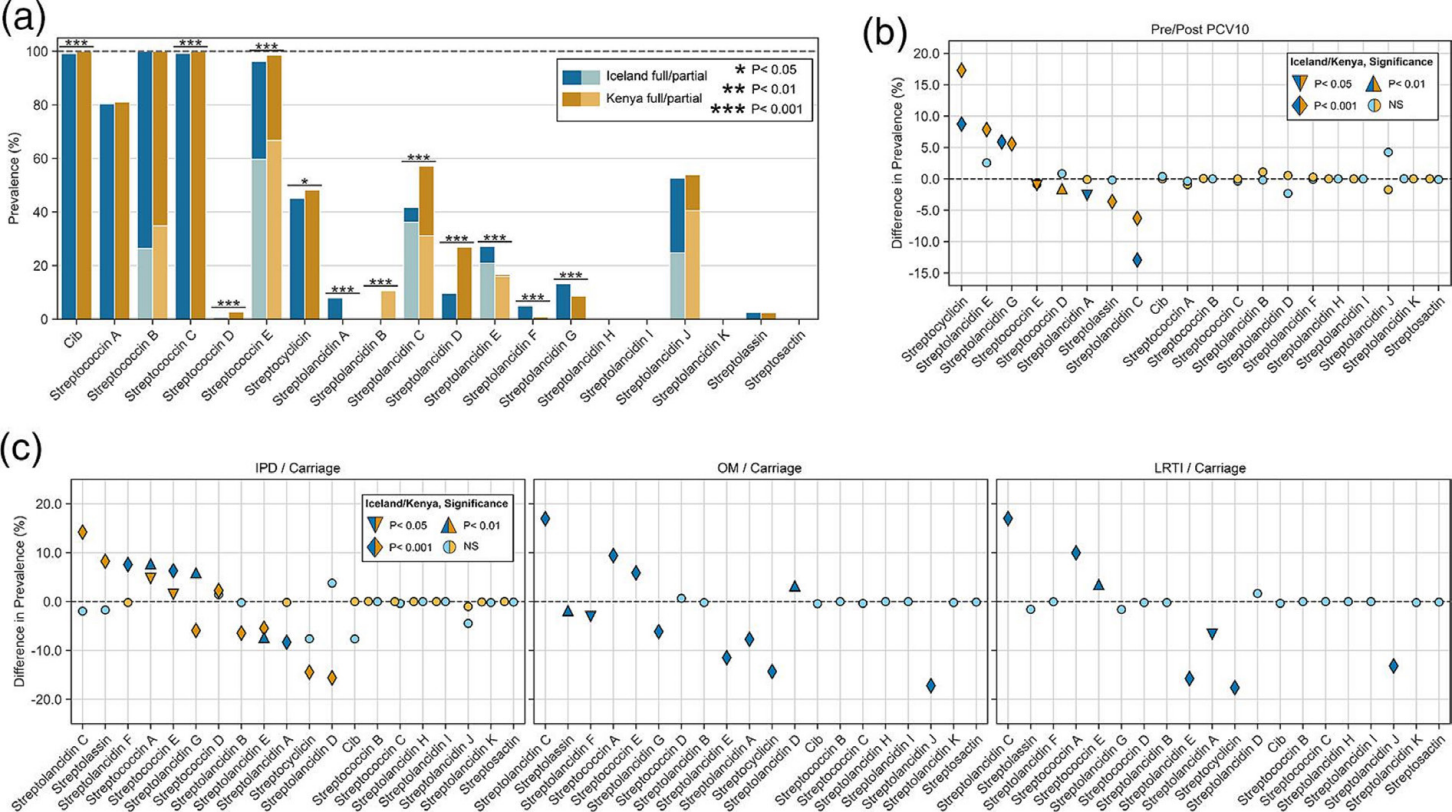
Prevalence of 19 different bacteriocin clusters in the Icelandic and Kenyan datasets. (**a**) Overall prevalence of each bacteriocin cluster in Iceland and Kenya. (**b**) Differences in prevalence of bacteriocin clusters in the post-PCV10 vs pre-PCV10 time periods. (**c**) Differences in the prevalence of bacteriocin clusters among invasive pneumococci (IPD) vs carriage pneumococci in Iceland and Kenya (left panel), pneumococci causing otitis media (OM) vs carriage pneumococci in Iceland (middle panel), and pneumococci causing lower respiratory tract infection (LRTI) vs carriage pneumococci in Iceland (right panel). Icelandic data are displayed with blue bars and symbols, and Kenyan data are displayed with tan bars and symbols. Significant differences were assessed in all cases using a Chi-square test. See Methods for definition of full and partial clusters.

Overall, cib and streptococcin B, C and E clusters were present in 96–100 % of pneumococci from both countries, whereas streptolancidin H and I clusters were never observed (Fig. 2a). The remaining bacteriocin clusters ranged in prevalence from 0.1 –81 % per dataset. Twelve bacteriocin clusters were significantly more common in one dataset than the other (Chi-square test, Table S6) but the largest differences in prevalence were among the streptolancidins (Fig. 2a). For example, 10.7 % (*n*=340 genomes) of Kenyan pneumococci harboured a streptolancidin B cluster as compared to just two Icelandic pneumococci (Fig. 2a; Table S7).

**Fig. 3.**
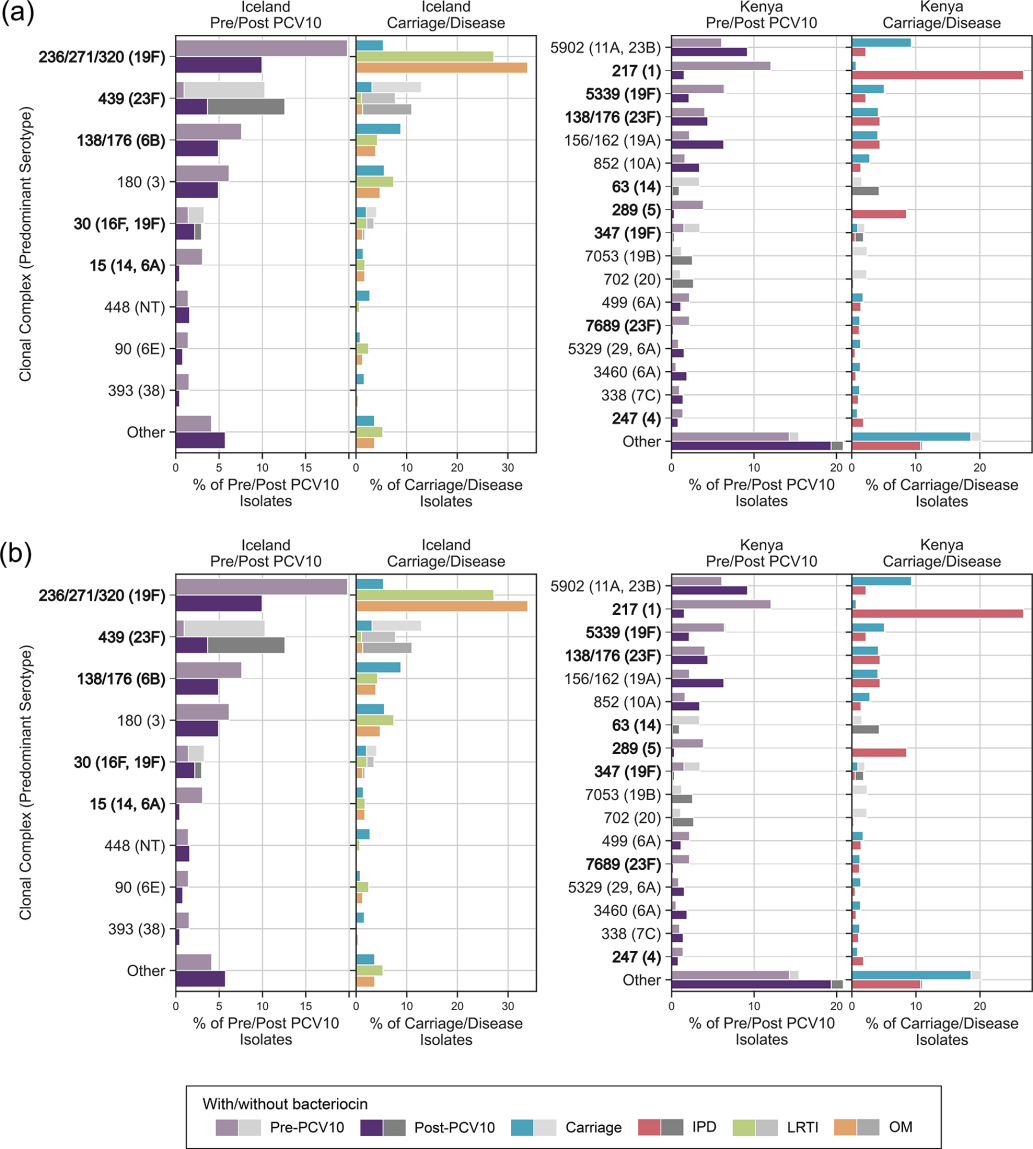
Prevalence of streptolancidin C (panel **a**) and streptocyclicin (panel **b**) bacteriocin clusters among pneumococci in two groups, carriage vs disease and pre- vs post-PCV10, stratified by clonal complex (CC). Each plot shows all CCs in which the bacteriocin cluster was detected. Any CC representing <1 % of the overall dataset was in the ‘Other’ category. Each bar represents the overall percentage of pneumococci within that CC, the coloured section of the bar represents those pneumococci with the bacteriocin cluster, and the grey section represents those without the bacteriocin cluster. For Icelandic disease-causing pneumococci, only the disease in which the bacteriocin cluster was significantly altered relative to carriage are shown. Note: the dominant serotype(s) of each CC is shown in brackets. PCV10 serotypes are in bold font. IPD, invasive pneumococcal disease; LRTI, lower respiratory tract infection; OM, otitis media.

### Bacteriocin prevalence was altered in the post-PCV10 period

Statistically significant differences in the prevalence of eight bacteriocin clusters were observed among pneumococci in the post-PCV10 period: streptococcins D, E, streptolancidins A, C, E, G, streptocyclicin, and streptolassin (Chi-square test, Table S6; Fig. 2b). Among Icelandic pneumococci the prevalence of streptocyclicin and streptolancidin G increased, and streptolancidins A and C decreased. Among Kenyan pneumococci, streptocyclicin and streptolancidins E and G increased in prevalence, and streptococcins E and D, streptolancidin C and streptolassin decreased in prevalence.

### Bacteriocin cluster prevalence differed among pneumococci from carriage and disease

A comparison of carriage and invasive Kenyan pneumococci revealed significant differences in the prevalence of ten bacteriocin clusters, five of which were more common among invasive pneumococci (streptolancidin C, streptolassin, streptococcins A, D and E), and five were more common among carriage pneumococci (streptolancidins B, D, E, G and streptocyclicin; Fig. 2c, left panel; Table S6). A range of serotypes were represented among invasive and carriage pneumococci (Table S8). Similarly, significant differences in the prevalence of bacteriocin clusters were observed among Icelandic pneumococci from disease and carriage, as shown in Fig. 2c (all three panels): IPD vs carriage, six bacteriocins (streptolancidins A, E, F, G, streptococcins A, E); OM vs carriage, 11 bacteriocins (streptolancidins A, C, D, E, F, G, J, streptococcins A, E, streptocyclicin, streptolassin; and LRTI vs carriage, seven bacteriocins (streptolancidins A, C, E, J, streptococcin A, E, streptocyclicin). Overall, streptococcin A and E clusters were significantly more common among Icelandic pneumococci recovered from all three disease processes relative to carriage pneumococci, and a range of serotypes were represented (Fig. 2c; Tables S6 and S8).

### Bacteriocin cluster prevalence and post-vaccine population restructuring

The observed differences in bacteriocin prevalence were investigated relative to changes in the frequency of CCs pre- and post-PCV10 implementation. For example, streptolancidin C clusters were significantly associated with pre-PCV10 pneumococci in both datasets (Fig. 2b; Table S6), specifically with pneumococci causing IPD in the Kenyan dataset, and OM and LRTI in the Icelandic dataset (Figs 2c and 3a). Streptolancidin C clusters were mostly identified within pneumococci of CCs associated with vaccine serotypes (1, 4, 5, 6B, 14, 19F, 23F) across the two countries (Fig. 3a).

In contrast, streptocyclicin clusters were significantly more prevalent post-PCV10 (Fig. 2b; Table S6), among carriage rather than disease pneumococci (Figs 2c and 3b), and were identified among a range of vaccine (5, 6B, 9V, 14, 18C, 19F, 23F) and nonvaccine serotype pneumococci (Fig. 3b). All of the bacteriocin clusters with significantly different pre- or post-PCV10 frequencies as shown in Fig. 2b were inspected, and corresponding increases or decreases in the frequency of CCs pre- and post-PCV10 introduction were generally found to explain the differences in bacteriocins (Tables S9 and S10).

### Bacteriocin repertoires shared between the two datasets and within lineages

**Fig. 4.**
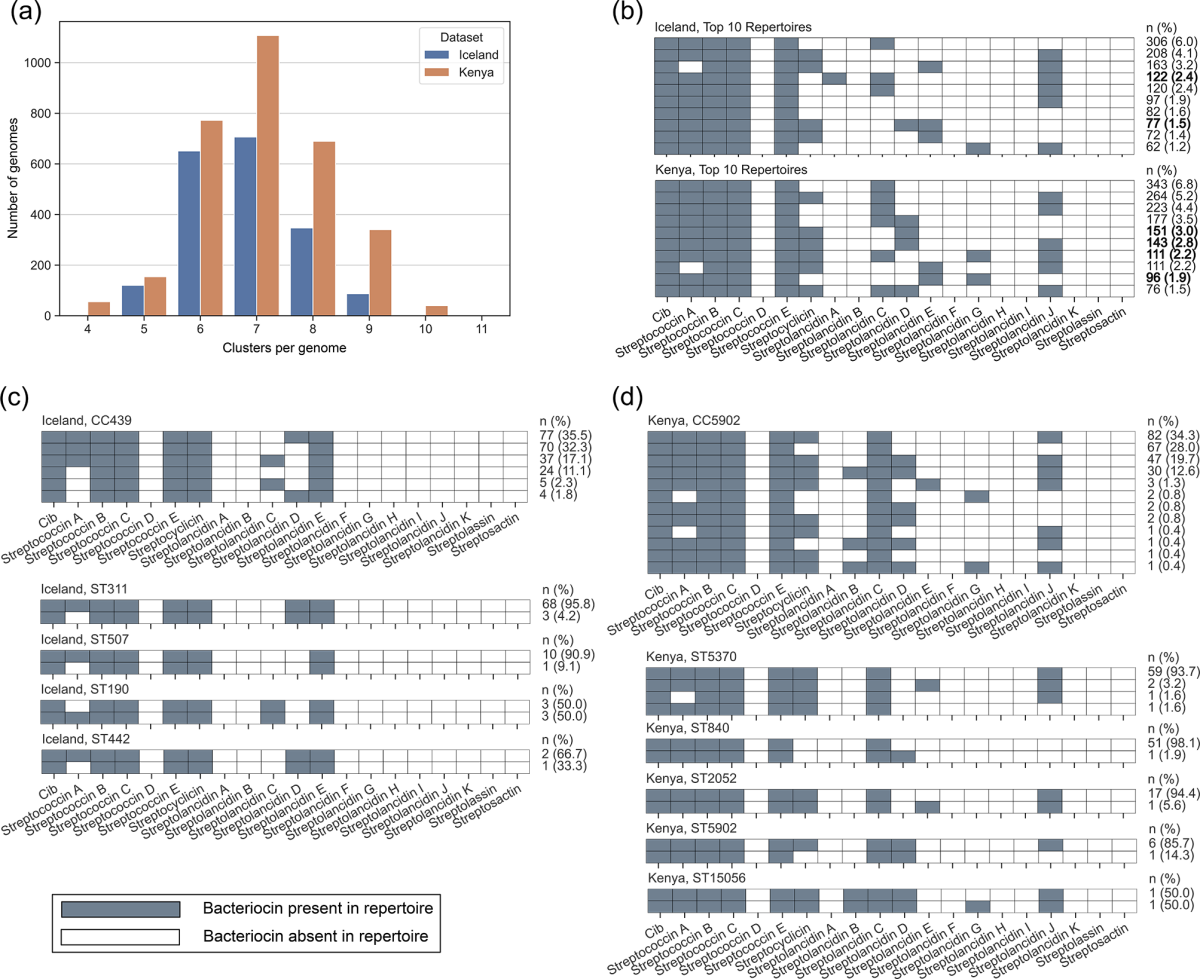
Bacteriocin repertoires observed among Icelandic and Kenyan pneumococci. (**a**) Number of bacteriocin clusters detected per genome. (**b**) The composition of the ten most frequently observed bacteriocin repertoires in Icelandic and Kenyan pneumococci. Bold text indicates that the repertoire was found only in that country. (**c, d**) Bacteriocin repertoires observed in genomes from CC439 in the Icelandic dataset (panel c), and CC5902 in the Kenyan dataset (panel d), including a more detailed breakdown of the CC by sequence type (ST) below the CC summary. STs with no differences in repertoire are not shown. Note: the frequency of each repertoire within the Icelandic or Kenyan dataset, respectively, is given at the far right of each diagram.

Finally, the combination of different bacteriocin clusters in each genome, or ‘repertoire’, was investigated. Overall, each pneumococcal genome harboured between four and 11 different bacteriocin clusters, and in both datasets the mode was seven (Fig. 4a). We found 89 and 81 % of genomes had between six and eight bacteriocin clusters in the Icelandic and Kenyan datasets, respectively. Overall, 134 different bacteriocin repertoires were observed, although more variation was observed in the Kenyan dataset (*n*=103 repertoires) than the Icelandic dataset (*n*=74 repertoires). In total, 43 repertoires were common to both datasets and the most frequently observed one included cib, streptococcins A, B, C, E, and streptolancidin C (Fig. 4b).

The bacteriocin repertoire was generally consistent among all pneumococci within the same CC; although there were examples of CCs (Iceland, *n*=29; Kenya, *n*=61) in which more than one bacteriocin repertoire was observed and some of the STs that comprised the CC also had variable repertoires, suggesting that there was movement of bacteriocin clusters among some pneumococci (Tables S11 and S12). For example, CC439 in Iceland (Fig. 4c) and CC5902 (Fig. 4d) in Kenya demonstrated minor differences in the presence or absence of bacteriocin clusters, and these differences were maintained even within some STs.

## Discussion

Bacteriocins are complicated in terms of their high prevalence and distribution among pneumococci, but there are also clear patterns emerging from this and previously published work that allow for more focused study of individual bacteriocin clusters, repertoires of bacteriocins, and the pneumococcal lineages that harbour them. In this study we observed significant differences in bacteriocin cluster prevalence between Icelandic and Kenyan pneumococci, carriage and disease pneumococci, and pneumococci recovered pre- and post-PCV10 introduction. These observations could largely be explained by different underlying pneumococcal population structures in each country, as bacteriocin clusters tended to be associated with specific CCs. Nevertheless, a bacteriocin repertoire was not necessarily fixed within a CC and there were many examples where genetically related pneumococci had gained or lost bacteriocin clusters. This would be consistent with horizontal gene transfer of whole bacteriocin clusters among pneumococci, either by homologous recombination, or within integrative conjugative elements (ICEs) [34–37].

These patterns are important because they provide the framework on which to propose hypotheses and experiments that allow for further study of these bacteriocins. It is also essential to understand any individual bacteriocin clusters in the context of all the other bacteriocin clusters present within the same genome, rather than in isolation, since every pneumococcal genome possesses many different bacteriocin clusters. Notably, our earlier study showed that multiple bacteriocin genes from different bacteriocin clusters were expressed simultaneously, but how the expression and regulation of these clusters is governed is still unknown [15].

An additional factor in this complexity is that some of these bacteriocin clusters are also detectable in genomes of non-pneumococcal *Streptococcus* spp such as *Streptococcus mitis, Streptococcus oralis* and *Streptococcus pseuodopneumoniae* [15]. Horizontal genetic exchange between pneumococci and non-pneumococcal streptococci has been documented since the 1990s and is a major driver in the evolution of these organisms [38–41]. Understanding bacteriocin-mediated competition between different *Streptococcus* species (and other nasopharyngeal colonisers) will also be required to fully understand microbial interactions in the nasopharynx.

Moreover, if pneumococcal bacteriocin clusters are horizontally exchanged among pneumococci and/or other streptococci, one consequence is that genetic lineages could adapt to altered competition dynamics in remodelled post-PCV populations by acquiring a bacteriocin repertoire that improves competitiveness, and this may (or may not) lead to increased pneumococcal disease. In this study, it was not possible to consider the dynamics of bacteriocins among co-colonising pneumococcal strains, nor could we investigate contributions from other commensal microbes that share the same ecological niche and compete for the same resources. Experimental studies of microbial competition would likely be helpful in this context.

Overall, this study focused on bacteriocins in two large, geographically distinct, well characterised pneumococcal populations, and revealed the prevalence and diversity of bacteriocins harboured by carriage and disease-causing pneumococci with greater clarity. This work also demonstrated that PCV-mediated perturbations in the pneumococcal population structure led to changes in the distribution of bacteriocin clusters, which may have consequences for pneumococcal disease. Further work will be required to establish the role of pneumococcal bacteriocins in nasopharyngeal competition and also the range of species targeted by these bacteriocins.

## Supplementary Data

Supplementary material 1

Supplementary material 2

Supplementary material 3

Supplementary material 4
