# Supplementary material for: Nasopharyngeal competition dynamics are likely to be altered following vaccine introduction: bacteriocin prevalence and diversity among Icelandic and Kenyan pneumococci
Source: Microb Genom. 2023 Jul 12;9(7):mgen001060. doi: 10.1099/mgen.0.001060 (PMC10438807; doi:10.1099/mgen.0.001060)
Supplement: Supplementary material 1 [file mgen-9-1060-s001.pdf]

Supplementary Materials

Nasopharyngeal competition dynamics are likely to be altered following vaccine introduction: bacteriocin prevalence and diversity among Icelandic and Kenyan pneumococci

Madeleine EB Butler[1], Melissa J Jansen van Rensburg[2], Angela Karani[3], Benedict Mvera[3], Donald Akech[3], Asma Akter[1], Calum Forrest[1], Andries J van Tonder[4], Sigríður J Quirk[5], Gunnsteinn Haraldsson[5], Stephen D Bentley[6], Helga Erlendsdóttir[5], Ásgeir Haraldsson[7], Karl G Kristinsson[5], J Anthony G Scott[3,8], Angela B Brueggemann[1,2]

[1]Imperial College London, London, United Kingdom
[2]University of Oxford, Oxford, United Kingdom
[3]KEMRI Wellcome Trust Programme, Kilifi, Kenya
[4]University of Cambridge, Cambridge, United Kingdom
[5]University of Iceland and Landspitali - The National University Hospital of Iceland, Reykjavík, Iceland
[6]Wellcome Sanger Institute, Hinxton, United Kingdom
[7]University of Iceland and Children's Hospital Iceland, Reykjavík, Iceland
[8]London School of Hygiene and Tropical Medicine, London, United Kingdom

Corresponding author:
Prof Angela Brueggemann
angela.brueggemann@ndph.ox.ac.uk

Supplementary data

Supplementary Table 1: Full, partial, and fragmented bacteriocin clusters identified among Icelandic and Kenyan pneumococci.

Bacteriocin	Country	Profile	Category	Frequency
Cib	Kenya	A-B-C	Full	3159
	Iceland	A-B-C	Full	1895
		/-/-C	Fragment	17
Streptococcin A	Kenya	A-B-C	Full	2562
	Iceland	A-B-C	Full	1537
Streptococcin B	Kenya	A-B-C	Full	2058
		/-B-C	Partial	1100
		/-B-/	Fragment	1
	Iceland	A-B-C	Full	1408
		/-B-C	Partial	504
Streptococcin C	Kenya	A-B-C	Full	3159
	Iceland	A-B-C	Full	1912
Streptococcin D	Kenya	A-B-C	Full	85
	Iceland	A-B-C	Full	9
Streptococcin E	Kenya	A-B-C	Full	1048
		/-B-C	Partial	2107
	Iceland	A-B-C	Full	699
		/-B-C	Partial	1142
Streptocyclicin	Kenya	A-B-C-D-E	Full	1524
		/-/-/-D-/	Fragment	1
	Iceland	A-B-C-D-E	Full	862
Streptolancidin A	Kenya	A1-A2-A3-A4-A5-F-E-K-R-M-T	Full	8
		/-/-/-/-/-/-/-/-/-/-T	Fragment	2
	Iceland	A1-A2-A3-A4-A5-F-E-K-R-M-T	Full	152
		/-/-/-/-/-/-/-/-/-/-T	Fragment	38
Streptolancidin B	Kenya	F-G-E-A-M-T	Full	2
		F-G-E-/-/-/	Partial	336
	Iceland	F-G-E-/-/-/	Partial	2
Streptolancidin C	Kenya	A-X-L-T	Full	826
		A-X-/-/	Partial	983
		/-/-/-T	Fragment	1
	Iceland	A-X-L-T	Full	106
		A-X-/-/	Partial	692
Streptolancidin D	Kenya	A-B-C-T	Full	853
	Iceland	A-B-C-T	Full	184
Streptolancidin E	Kenya	M1-A1-A2-M2-M3-T-X1-F-G-X2	Full	29
		/-/-/-/-M3-T-X1-F-G-X2	Partial	493

		/-/-/-/-M3-T-/-F-G-X2	Partial	10
	Iceland	M1-A1-A2-M2-M3-T-X1-F-G-X2	Full	142
		/-/-/-/-M3-T-X1-F-G-X2	Partial	309
		/-/-/-/-M3-T-/-F-G-X2	Partial	91
Streptolancidin F	Kenya	A-L	Full	25
	Iceland	A-L	Full	95
Streptolancidin G	Kenya	A1-A2-M-D-P1-T-P2	Full	272
	Iceland	A1-A2-M-D-P1-T-P2	Full	252
Streptolancidin J	Kenya	A1-L-P-T1-T2-T3-A2	Full	426
		A1-L-P-T1-T2-T3-/	Partial	1
		A1-L-/-T1-T2-T3-A2	Partial	931
		A1-L-/-T1-T2-T3-/	Partial	340
		A1-/-/-/-/-/-/	Fragment	2
		/-L-P-T1-T2-T3-A2	Partial	1
		/-L-/-T1-T2-T3-A2	Partial	6
		/-L-/-T1-T2-T3-/	Partial	1
		/-/-/-/-/-T3-/	Fragment	1
	Iceland	A1-L-P-T1-T2-T3-A2	Full	535
		A1-L-P-T1-T2-T3-/	Partial	40
		A1-L-/-T1-T2-T3-A2	Partial	383
		A1-L-/-T1-T2-T3-/	Partial	30
		A1-L-/-/-T2-T3-A2	Partial	1
		/-L-P-T1-T2-T3-A2	Partial	12
		/-L-/-T1-T2-T3-A2	Partial	6
Streptolancidin K	Kenya	A-L-T	Full	2
		/-/-T	Fragment	3
	Iceland	A-L-T	Full	2
		/-/-T	Fragment	5
Streptolassin	Kenya	A-C-B1-B2-F-E-G-R-K	Full	77
	Iceland	A-C-B1-B2-F-E-G-R-K	Full	48
Streptosactin	Iceland	A-CD-X1-X2-P-X3	Full	1

Note: Rows shaded in grey indicate fragmented clusters, which were excluded from further analysis.

Genome assemblies and isolate provenance data are publicly available via PubMLST:
https://pubmlst.org/bigsdb?db=pubmlst_spneumoniae_isolates_all

Supplementary Table 2: Icelandic genome and provenance data

Supplementary Table 3: Kenyan genome and provenance data

Supplementary Table 4: Genes associated with bacteriocin clusters in the Icelandic and Kenyan datasets.

Bacteriocin	Bacteriocin type	Gene	Gene aliases	GenBank accession no.	Typical length (bp) [a]	Predicted functionality
Cib[b,c]	Competence-induced	*cibA*		NC_003028.3	186	Bacteriocin precursor
		cibB			153	Bacteriocin precursor
		cibC			198	Immunity
Streptococcin A[b]	Lactococcin 972-like	*scaA*		MF990778.1	285	Bacteriocin precursor
		scaB			2109	Immunity
		scaC			642	Immunity
Streptococcin B[b]	Lactococcin 972-like	*scbA*		MF990779.1	297	Bacteriocin precursor
		scbB			2031	Immunity
		scbC			642	Immunity
Streptococcin C[b]	Lactococcin 972-like	*sccA*		MF990780.1	348	Bacteriocin precursor
		sccB			2022	Immunity
		sccC			642	Immunity
Streptococcin D[b]	Lactococcin 972-like	*scdA*		MF990781.1	297	Bacteriocin precursor
		scdB			2010	Immunity
		scdC			633	Immunity
Streptococcin E[b]	Lactococcin 972-like	*sceA*		MF990782.1	297	Bacteriocin precursor
		sceB			2016	Immunity
		sceC			633	Immunity
Streptocyclicin [b,d]	Head-to-tail cyclised peptides	*scyA*		MF990796.1	297	Bacteriocin precursor
		scyB			1137	Bacteriocin biosynthesis
		scyC			483	Bacteriocin biosynthesis
		scyD			597	Bacteriocin biosynthesis
		scyE			492	Bacteriocin biosynthesis
Streptolancidin A[b,e,f]	Class II lanthipeptide	*slaA1*	*pldA1-4, srnA*	MF990783.1	183	Bacteriocin precursor
		slaA2			183	Bacteriocin precursor
		slaA3			183	Bacteriocin precursor
		slaA4			177	Bacteriocin precursor
		slaA5			108	Bacteriocin precursor
		slaF	*pldF. srnX*		738	Immunity
		slaE	*pldE, srnY*		2016	Immunity
		slaK	*pldK, srnK*		1575	Histidine kinase/response regulator
		slaR	*pldR, srnR*		597	Histidine kinase/response regulator
		slaM	*pldM, srnM*		2955	Bacteriocin biosynthesis
		slaT	*pldT, srnT*		2124	Transporter
Streptolancidin B[b,g]	Class II lanthipeptide	*slbF*		MF990784.1	936	Immunity
		slbG			741	Immunity
		slbE			729	Immunity

		slbA	*lcpA*		216	Bacteriocin precursor
		slbM	*lcpM*		3252	Bacteriocin biosynthesis
		slbT	*lcpT*		2067	Transporter
Streptolancidin C[b]	Class IV lanthipeptide	*slcA*		MF990785.1	105	Bacteriocin precursor
		slcX			993	Unknown
		slcL			1437	Bacteriocin biosynthesis
		slcT			1239	Transporter
Streptolancidin D[b]	Class I lanthipeptide	*sldA*		MF990786.1	105	Bacteriocin precursor
		sldB			2793	Bacteriocin biosynthesis
		sldC			1278	Bacteriocin biosynthesis
		sldT			1257	Transporter
Streptolancidin E[b,h]	Class II lanthipeptide	*sleM1*		MF990787.1	3036	Bacteriocin biosynthesis
		sleA1			171	Bacteriocin precursor
		sleA2			192	Bacteriocin precursor
		sleM2			2016	Bacteriocin biosynthesis
		sleM3			747	Bacteriocin biosynthesis
		sleT			2142	Transporter
		sleX1			171	Unknown
		sleF			729	Immunity
		sleG			738	Immunity
		sleX2			711	Unknown
Streptolancidin F[b]	Class IV lanthipeptide	*slfA*		MF990788.1	99	Bacteriocin precursor
		slfL			2496	Bacteriocin biosynthesis
Streptolancidin G[b,i]	Class II lanthipeptide	*slgA1*		MF990789.1	225	Bacteriocin precursor
		slgA2			189	Bacteriocin precursor
		slgM			2991	Bacteriocin biosynthesis
		slgD			705	Bacteriocin biosynthesis
		slgP1			930	Bacteriocin biosynthesis
		slgT			2109	Transporter
		slgP2			1740	Bacteriocin biosynthesis
Streptolancidin H[b]	Class I lanthipeptide	*slhP*		MF990790.1	1368	Bacteriocin biosynthesis
		slhR			684	Histidine kinase/response regulator
		slhK			1341	Histidine kinase/response regulator
		slhF			687	Immunity
		slhE			741	Immunity
		slhG			678	Immunity
		slhX1			645	Unknown
		slhX2			285	Unknown
		slhA			177	Bacteriocin precursor
		slhB			2964	Bacteriocin biosynthesis
		slhT			1776	Transporter

		slhC			1272	Bacteriocin biosynthesis
		slhI			663	Immunity
Streptolancidin I[b]	Class I lanthipeptide	*sliP*		MF990791.1	1374	Bacteriocin biosynthesis
		sliR			699	Histidine kinase/response regulator
		sliK			1344	Histidine kinase/response regulator
		sliF			702	Immunity
		sliE			738	Immunity
		sliG			687	Immunity
		sliA			168	Bacteriocin precursor
		sliB			2976	Bacteriocin biosynthesis
		sliT			1809	Transporter
		sliC			1278	Bacteriocin biosynthesis
		sliI			717	Immunity
Streptolancidin J[b]	Class IV lanthipeptide	*sljA1*		MF990792.1	138	Bacteriocin precursor
		sljL			2610	Bacteriocin biosynthesis
		sljP			1941	Bacteriocin biosynthesis
		sljT1			1608	Transporter
		sljT2			741	Transporter
		sljT3			1320	Transporter
		sljA2			138	Bacteriocin precursor
Streptolancidin K[b]	Class IV lanthipeptide	*slkA*		MF990793.1	99	Bacteriocin precursor
		slkL			2517	Bacteriocin biosynthesis
		slkT			1221	Transporter
Streptolassin[b]	Lasso peptide	*slsA*		MF990794.1	129	Bacteriocin precursor
		slsC			1725	Bacteriocin biosynthesis
		slsB1			252	Bacteriocin biosynthesis
		slsB2			2232	Bacteriocin biosynthesis
		slsF			717	Immunity
		slsE			792	Immunity
		slsG			711	Immunity
		slsR			774	Histidine kinase/response regulator
		slsK			1098	Histidine kinase/response regulator
Streptosactin[b]	Sactipeptide	*ssaA*		MF990795.1	177	Bacteriocin precursor
		ssaCD			1338	Bacteriocin biosynthesis
		ssaX1			153	Unknown
		ssaX2			996	Unknown
		ssaP			861	Bacteriocin biosynthesis
		ssaX3			696	Unknown

a. Length of genes as published previously (see b)

b. Rezaei Javan R, van Tonder AJ, King JP, Harrold CL, Brueggemann AB. Genome sequencing reveals a large and diverse repertoire of antimicrobial peptides. *Front Microbiol*. 2018;9(AUG):1-15. doi:10.3389/fmicb.2018.02012

c. Guiral S, Mitchell TJ, Martin B, Claverys JP. Competence-programmed predation of noncompetent cells in the human pathogen *Streptococcus pneumoniae*: Genetic requirements. *Proc Natl Acad Sci U S A*. 2005;102(24):8710-8715. doi:10.1073/pnas.0500879102

d. Bogaardt C, van Tonder AJ, Brueggemann AB. Genomic analyses of pneumococci reveal a wide diversity of bacteriocins - including pneumocyclicin, a novel circular bacteriocin. *BMC Genomics*. 2015;16(1):554. doi:10.1186/s12864-015-1729-4

e. Maricic N, Anderson ES, Opipari AME, Yu EA, Dawid S. Characterization of a multipeptide lantibiotic locus in *Streptococcus pneumoniae*. *mBio*. 2016;7(1):1656-1671. doi:10.1128/mBio.01656-15

f. Walker G V., Heng NCK, Carne A, Tagg JR, Wescombe PA. Salivaricin E and abundant dextranase activity may contribute to the anti-cariogenic potential of the probiotic candidate *Streptococcus salivarius* JH. *Microbiol U K*. 2016;162(3):476-486. doi:10.1099/mic.0.000237

g. Kadam A, Eutsey RA, Rosch J, et al. Promiscuous signaling by a regulatory system unique to the pandemic PMEN1 pneumococcal lineage. *PLoS Pathog*. 2017;13(5). doi:10.1371/journal.ppat.1006339

h. Begley M, Cotter PD, Hill C, Ross RP. Identification of a novel two-peptide lantibiotic, lichenicidin, following rational genome mining for LanM proteins. *Appl Environ Microbiol*. 2009;75(17):5451-5460. doi:10.1128/AEM.00730-09

i. Hoover SE, Perez AJ, Tsui HCT, et al. A new quorum-sensing system (TprA/PhrA) for *Streptococcus pneumoniae* D39 that regulates a lantibiotic biosynthesis gene cluster. *Mol Microbiol*. 2015;97(2):229-243. doi:10.1111/mmi.1302

Supplementary Table 5: Full and partial bacteriocin clusters identified among Icelandic and Kenyan pneumococci.

**Iceland**			
**Bacteriocin cluster**	**Category**	**Frequency**	**% of total bacteriocin clusters**
Cib	Contiguous	1895	100
Streptococcin A	Contiguous	1537	100
Streptococcin B	Contiguous	1912	100
Streptococcin C	Contiguous	1811	94.72
	EOC	82	4.29
	Non-contiguous (multiple contigs, not EOC-adjacent)	16	0.84
	Contiguous with Ns	3	0.16
Streptococcin D	Contiguous	9	100
Streptococcin E	Contiguous	1840	99.95
	Non-contiguous (multiple contigs, not EOC-adjacent)	1	0.05
Streptocyclicin	Contiguous	860	99.77
	EOC	2	0.23
Streptolancidin A	Contiguous	152	100
Streptolancidin B	Contiguous	2	100
Streptolancidin C	Contiguous	798	100
Streptolancidin D	Contiguous	183	99.46
	EOC	1	0.54
Streptolancidin E	Contiguous	461	85.06
	EOC	47	8.67
	Non-contiguous (multiple contigs, not EOC-adjacent)	15	2.77
	Contiguous with Ns	13	2.40
	Non-contiguous (multiple contigs, non-adjacent loci)	6	1.11
Streptolancidin F	Contiguous	95	100
Streptolancidin G	Contiguous	252	100
Streptolancidin J	Contiguous	1005	99.80
	Non-contiguous (one contig)	2	0.20
Streptolancidin K	Contiguous	2	100
Streptolassin	Contiguous	48	100
Streptosactin	Contiguous	1	100

Kenya			
**Bacteriocin cluster**	**Category**	**Frequency**	**% of total bacteriocin clusters**
Cib	Contiguous	3159	100
Streptococcin A	Contiguous	2559	99.88
	Non-contiguous (multiple contigs, non-adjacent loci)	3	0.12
Streptococcin B	Contiguous	3157	99.97
	EOC	1	0.03
Streptococcin C	Contiguous	3152	99.78
	EOC	7	0.22
Streptococcin D	Contiguous	85	100
Streptococcin E	Contiguous	3099	98.23
	Non-contiguous (multiple contigs, non-adjacent loci)	26	0.82
	EOC	16	0.51
	Non-contiguous (one contig)	8	0.25
	Non-contiguous (multiple contigs, not EOC-adjacent)	6	0.19
Streptocyclicin	Contiguous	1516	99.48
	EOC	7	0.46
	Non-contiguous (multiple contigs, not EOC-adjacent)	1	0.07
Streptolancidin A	Contiguous	8	100
Streptolancidin B	Contiguous	338	100
Streptolancidin C	Contiguous	1767	97.68
	EOC	38	2.10
	Non-contiguous (multiple contigs, non-adjacent loci)	3	0.17
	Non-contiguous (multiple contigs, not EOC-adjacent)	1	0.06
Streptolancidin D	Contiguous	850	99.65
	Non-contiguous (multiple contigs, non-adjacent loci)	1	0.12
	EOC	1	0.12
	Non-contiguous (multiple contigs, not EOC-adjacent)	1	0.12
Streptolancidin E	Contiguous	518	97.37
	EOC	8	1.50

	Non-contiguous (multiple contigs, not EOC-adjacent)	4	0.75
	Non-contiguous (multiple contigs, non-adjacent loci)	1	0.19
	Contiguous with Ns	1	0.19
Streptolancidin F	Contiguous	25	100
Streptolancidin G	Contiguous	272	100
Streptolancidin J	Contiguous	1691	99.12
	EOC	6	0.35
	Contiguous with Ns	5	0.29
	Non-contiguous (multiple contigs, not EOC-adjacent)	3	0.18
	Non-contiguous (multiple contigs, non-adjacent loci)	1	0.06
Streptolancidin K	Contiguous	2	100
Streptolassin	Contiguous	77	100

Note: Bacteriocin clusters were categorised according to the proximity of the constituent genes to one another. Any clusters with an intergenic region >2.5kbp were categorised as non-contiguous. Bacteriocin clusters with genes on multiple contigs were categorised as 'end of contig' (EOC) if the genes were found within 2.5kbp of each other and the end of the contig, otherwise the clusters were categorised as non-contiguous (ie present on multiple contigs). Rows in grey represent non-contiguous clusters, which were excluded from further analysis.

Supplementary Table 6: Results of statistical analyses comparing the prevalence of bacteriocins in each of the two study datasets.

Supplementary Table 7: Streptolancidin clusters present in significantly different frequencies among Icelandic and Kenyan pneumococci, stratified by clonal complex (CC).

Number of pneumococci harbouring each streptolancidin cluster n (% of CC representatives in each dataset with the bacteriocin)		
**Streptolancidin A**		
**CC**	**Iceland**	**Kenya**
CC138/176	122 (100)	1 (0.8)
CC448	29 (100)	2 (100)
CC802	0	5 (100)
CC338	1 (12.5)	0
**Streptolancidin B**		
**CC**	**Iceland**	**Kenya**
CC702	0	57 (98.3)
CC499	0	55 (100)
CC5902	0	32 (13.4)
Sing11162	0	23 (100)
CC347	0	18 (29.0)
CC5250/5947/15006	0	18 (100)
CC703	0	16 (100)
CC385	0	13 (41.9)
CC1264	0	11 (100)
CC6446/14764	0	11 (100)
Other CCs	2 (100)	62 (34.6)
Other Singletons	0	22 (100)
**Streptolancidin C**		
**CC**	**Iceland**	**Kenya**
CC236/271/320	293 (100)	4 (100)
CC138/176	122 (100)	133 (100)
CC5902	0	239 (100)
CC217	0	223 (100)
CC5339	0	138 (97.2)
CC156/162	0	131 (100)
CC180	107 (100)	6 (100)
CC852	0	78 (100)
CC289	0	69 (100)
CC499	0	53 (96.4)
Other CCs	262 (52.6)	652 (71.8)
Other Singletons	14 (100)	79 (92.9)
**Streptolancidin D**		
**CC**	**Iceland**	**Kenya**
CC701	0	161 (98.8)
CC5339	0	139 (97.9)

CC991	0	104 (100)
CC5902	0	83 (34.7)
CC439	81 (37.3)	0
CC854	0	57 (100)
CC706	0	37 (100)
CC15	36 (100)	0
CC14774	0	23 (100)
Sing11162	0	23 (100)
Other CCs	55 (25.0)	190 (42.6)
Other Singletons	12 (100)	34 (100)
**Streptolancidin E**		
**CC**	**Iceland**	**Kenya**
CC439	217 (100)	0
CC199	174 (97.2)	0
CC1146	0	99 (71.2)
CC230	3 (100)	88 (95.7)
CC5258	0	76 (98.7)
CC1381	0	49 (100)
CC344	37 (100)	1 (100)
CC705/14790	0	38 (100)
CC448	29 (100)	2 (100)
CC138/176	0	22 (16.5)
Other CCs	59 (43.4)	115 (29.0)
Other Singletons	2 (100)	37 (100)
**Streptolancidin F**		
**CC**	**Iceland**	**Kenya**
CC344	33 (89.2)	1 (100)
CC100	25 (100)	0
CC191	16 (100)	0
CC5560/6090/6103	0	14 (100)
CC433	9 (14.8)	0
CC5292	0	7 (100)
CC717	4 (100)	0
CC97	3 (3.4)	0
CC346	2 (100)	0
CC113	2 (9.5)	0
Other CCs	0	2 (2.2)
Other Singletons	1 (100)	1 (100)
**Streptolancidin G**		
**CC**	**Iceland**	**Kenya**
CC1146	0	134 (96.4)
CC852	0	78 (100)

CC433	61 (100)	0
CC392	47 (100)	0
CC5329	0	37 (97.4)
CC393	20 (100)	9 (100)
CC66	18 (94.7)	0
CC30	17 (28.3)	0
CC2755	16 (100)	0
CC315	13 (86.7)	0
Other CCs	48 (63.2)	12 (4.0)
Other Singletons	12 (100)	2 (100)

Note: The 10 CCs with the biggest contribution to the frequency of each streptolancidin are shown. Other CCs were pooled to the 'Other' categories.

Supplementary Table 8: Association of bacteriocin clusters with pneumococcal serotype.

Streptococcin A			
Iceland		Kenya	
Significant in IPD, OM, LRTI		Significant in IPD	
Serotype	n (% of all pneumococci with that serotype)	Serotype	n (% of all pneumococci with that serotype)
19F	322 (97.3)	1	223 (99.6)
6A	163 (100)	19F	211 (92.5)
23F	151 (83.9)	6A	194 (94.2)
6B	121 (99.2)	19A	153 (100)
3	107 (98.2)	35B	139 (100)
11A	93 (100)	15A	136 (100)
14	81 (90.0)	15BC	130 (89.0)
22F	61 (100)	11A	116 (99.1)
23B	47 (100)	13	97 (99.0)
23A	45 (88.2)	14	95 (72.5)
Other serotypes	346 (61.3)	Other serotypes	1065 (68.9)
**Streptococcin D**			
Iceland		Kenya	
Not significant		Significant in IPD	
Serotype	n (% of all pneumococci with that serotype)	Serotype	n (% of all pneumococci with that serotype)
-	-	14	70 (53.4)
-	-	nontypable	15 (46.9)
**Streptococcin E**			
Iceland		Kenya	
Significant in IPD, OM, LRTI		Significant in IPD	
Serotype	n (% of all pneumococci with that serotype)	Serotype	n (% of all pneumococci with that serotype)
19F	328 (99.1)	19F	228 (100)
23F	180 (100)	1	224 (100)
6A	163 (100)	6A	206 (100)
19A	145 (100)	19A	153 (100)
6B	122 (100)	15BC	146 (100)
3	109 (100)	35B	139 (100)
11A	93 (100)	15A	136 (100)
15BC	93 (100)	14	131 (100)
14	90 (100)	6E(6Bii)	131 (100)

22F	61 (100)	23F	119 (100)
Other serotypes	456 (86.9)	Other serotypes	1502 (97.2)
**Streptocyclicin**			
**Iceland**		**Kenya**	
**Significant in carriage**		**Significant in carriage**	
**Serotype**	**n (% of all pneumococci with that serotype)**	**Serotype**	**n (% of all pneumococci with that serotype)**
23F	174 (96.7)	19A	147 (96.1)
19A	127 (87.6)	15A	126 (92.6)
15BC	93 (100)	6A	125 (60.7)
6A	69 (42.3)	13	98 (100)
14	66 (73.3)	11A	94 (80.3)
23A	50 (98.0)	16F	93 (96.9)
23B	43 (91.5)	23B	90 (100)
nontypable	36 (51.4)	34	85 (95.5)
9V	32 (100)	10A	82 (100)
16F	26 (100)	5	69 (100)
Other serotypes	146 (25.0)	Other serotypes	514 (29.8)
**Streptolancidin A**			
**Iceland**		**Kenya**	
**Significant in carriage**		**Not significant**	
**Serotype**	**n (% of all pneumococci with that serotype)**	**Serotype**	**n (% of all pneumococci with that serotype)**
6B	120 (98.4)	-	-
nontypable	29 (41.4)	-	-
6A	3 (1.8)	-	-
**Streptolancidin B**			
**Iceland**		**Kenya**	
**Not significant**		**Significant in carriage**	
**Serotype**	**n (% of all pneumococci with that serotype)**	**Serotype**	**n (% of all pneumococci with that serotype)**
-	-	6A	64 (31.1)
-	-	20	53 (98.1)
-	-	15BC	47 (32.2)
-	-	11A	33 (28.2)
-	-	16F	32 (33.3)
-	-	19F	23 (10.1)
-	-	6E(6Bii)	20 (15.3)

-	-	15A	12 (8.8)
-	-	24F	12 (100)
-	-	6C	10 (100)
-	-	Other serotypes	32 (5.1)
**Streptolancidin C**			
**Iceland**		**Kenya**	
**Significant in OM, LRTI**		**Significant in IPD**	
**Serotype**	**n (% of all pneumococci with that serotype)**	**Serotype**	**n (% of all pneumococci with that serotype)**
19F	325 (98.2)	1	224 (100)
6B	122 (100)	19F	180 (78.9)
3	109 (100)	19A	149 (97.4)
23A	40 (78.4)	6A	119 (57.8)
6A	29 (17.8)	23F	119 (100)
nontypable	29 (41.4)	11A	112 (95.7)
6E	24 (92.3)	15BC	88 (60.3)
14	21 (23.3)	23B	87 (96.7)
38	20 (100)	10A	81 (98.8)
7F	16 (100)	5	69 (100)
Other Serotypes	63 (10.8)	Other serotypes	577 (37.9)
**Streptolancidin D**			
**Iceland**		**Kenya**	
**Significant in OM**		**Significant in carriage**	
**Serotype**	**n (% of all pneumococci with that serotype)**	**Serotype**	**n (% of all pneumococci with that serotype)**
23F	83 (46.1)	19F	150 (65.8)
6A	22 (13.5)	15A	124 (91.2)
14	21 (23.3)	15BC	113 (77.4)
19F	17 (5.1)	13	97 (99.0)
35B	16 (48.5)	11A	75 (64.1)
19A	12 (8.3)	6E(6Bii)	57 (43.5)
6C	8 (27.6)	9V	51 (81.0)
18C	2 (9.1)	21	25 (40.3)
31	1 (33.3)	6A	22 (10.7)
6E	1 (3.8)	6B	21 (80.8)
Other serotypes	1 (12.5)	Other serotypes	116 (13.5)
**Streptolancidin E**			
**Iceland**		**Kenya**	
**Significant in carriage**		**Significant in carriage**	

Serotype	n (% of all pneumococci with that serotype)	Serotype	n (% of all pneumococci with that serotype)
23F	127 (70.6)	35B	96 (69.1)
19A	126 (86.9)	34	76 (85.4)
nontypable	67 (95.7)	16F	57 (59.4)
15BC	54 (58.1)	3	56 (62.2)
23A	50 (98.0)	18C	48 (100)
23B	42 (89.4)	14	34 (26.0)
18C	17 (77.3)	17F	20 (80.0)
9N	14 (77.8)	21	20 (32.3)
21	4 (13.8)	35F	17 (94.4)
1	3 (60.0)	15BC	14 (9.6)
Other serotypes	17 (2.6)	Other serotypes	89 (9.4)
**Streptolancidin F**			
**Iceland**		**Kenya**	
**Significant in IPD, carriage (vs OM)**		**Not significant**	
**Serotype**	**n (% of all pneumococci with that serotype)**	**Serotype**	**n (% of all pneumococci with that serotype)**
NT	34 (48.6)	-	-
33F	29 (100)	-	-
7F	16 (100)	-	-
22F	9 (14.8)	-	-
10A	3 (60.0)	-	-
19A	2 (1.4)	-	-
18C	2 (9.1)	-	-
**Streptolancidin G**			
**Iceland**		**Kenya**	
**Significant in IPD, carriage (vs OM)**		**Significant in carriage**	
**Serotype**	**n (% of all pneumococci with that serotype)**	**Serotype**	**n (% of all pneumococci with that serotype)**
22F	60 (98.4)	35B	134 (96.4)
23F	48 (26.7)	10A	82 (100)
35B	30 (90.9)	29	19 (67.9)
38	20 (100)	6A	12 (5.8)
6C	20 (69.0)	38	8 (34.8)
9N	18 (100)	6E(6Bii)	4 (3.1)
19F	17 (5.1)	10F	3 (33.3)

6A	14 (8.6)	15BC	2 (1.4)
19A	12 (8.3)	21	2 (3.2)
4	7 (100)	34	1 (1.1)
Other serotypes	6 (10.3)	Other serotypes	5 (1.5)
**Streptolancidin J**			
**Iceland**		**Kenya**	
**Significant in carriage**		**Not significant**	
**Serotype**	**n (% of all pneumococci with that serotype)**	**Serotype**	**n (% of all pneumococci with that serotype)**
6A	147 (90.2)	-	-
19A	139 (95.9)	-	-
6B	122 (100)	-	-
3	107 (98.2)	-	-
14	69 (76.7)	-	-
15BC	62 (66.7)	-	-
22F	61 (100)	-	-
23F	50 (27.8)	-	-
19F	34 (10.3)	-	-
9V	31 (96.9)	-	-
Other serotypes	183 (35.6)	-	-
**Streptolassin**			
**Iceland**		**Kenya**	
**Significant in carriage (v OM)**		**Significant in IPD**	
**Serotype**	**n (% of all pneumococci with that serotype)**	**Serotype**	**n (% of all pneumococci with that serotype)**
23F	48 (26.7)	5	69 (100)
-	-	37	3 (100)
-	-	7F	1 (50.0)
-	-	1	1 (0.4)
-	-	38	1 (4.3)
-	-	6A	1 (0.5)
-	-	8	1 (4.8)

Note: Up to 10 of the most common serotypes associated with each bacteriocin are listed separately, and the remainder were pooled as "Other". Bacteriocins that did not exhibit significantly altered prevalence in any subset of the data were excluded from this table.

Supplementary Table 9: Bacteriocin clusters in the Icelandic dataset.

Number of pneumococci harbouring each bacteriocin cluster, stratified by CC<br>n (% of CC representatives in each subset with the bacteriocin)						
**Streptococcin A**						
**CC**	**Pre-PCV**	**Post-PCV**	**Carriage**	**IPD**	**LRTI**	**OM**
CC236/271/320	201 (97.6)	85 (97.7)	53 (100)	6 (100)	72 (93.5)	155 (98.7)
CC439	86 (80.4)	98 (89.1)	106 (83.5)	16 (94.1)	21 (95.5)	41 (80.4)
CC138/176	79 (100)	42 (97.7)	87 (100)	5 (100)	11 (91.7)	18 (100)
CC180	64 (100)	42 (97.7)	55 (100)	9 (100)	21 (100)	21 (95.5)
CC62	37 (97.4)	56 (100)	62 (100)	5 (100)	13 (92.9)	13 (100)
CC490	40 (100)	34 (100)	46 (100)	5 (100)	9 (100)	14 (100)
CC433	13 (100)	48 (100)	32 (100)	13 (100)	11 (100)	5 (100)
CC30	34 (100)	26 (100)	40 (100)	2 (100)	10 (100)	8 (100)
CC97	30 (88.2)	30 (56.6)	32 (66.7)	4 (66.7)	4 (44.4)	20 (83.3)
CC124	36 (81.8)	17 (94.4)	23 (79.3)	12 (100)	5 (71.4)	13 (92.9)
Other CCs	213 (91.8)	211 (96.8)	200 (91.7)	75 (93.8)	65 (98.5)	84 (97.7)
Other Singletons	4 (100)	11 (100)	10 (100)	1 (100)	1 (100)	3 (100)
**Streptococcin E**						
**CC**	**Pre-PCV**	**Post-PCV**	**Carriage**	**IPD**	**LRTI**	**OM**
CC236/271/320	203 (98.5)	87 (100)	53 (100)	6 (100)	75 (97.4)	156 (99.4)
CC439	107 (100)	110 (100)	127 (100)	17 (100)	22 (100)	51 (100)
CC199	99 (100)	80 (100)	110 (100)	13 (100)	11 (100)	45 (100)
CC138/176	79 (100)	43 (100)	87 (100)	5 (100)	12 (100)	18 (100)
CC180	64 (100)	43 (100)	55 (100)	9 (100)	21 (100)	22 (100)
CC62	38 (100)	56 (100)	62 (100)	5 (100)	14 (100)	13 (100)
CC97	34 (100)	53 (100)	48 (100)	6 (100)	9 (100)	24 (100)
CC490	40 (100)	34 (100)	46 (100)	5 (100)	9 (100)	14 (100)
CC124	44 (100)	18 (100)	29 (100)	12 (100)	7 (100)	14 (100)
CC433	13 (100)	48 (100)	32 (100)	13 (100)	11 (100)	5 (100)
Other CCs	279 (99.6)	253 (100)	262 (99.6)	91 (100)	83 (100)	96 (100)
Other Singletons	4 (100)	11 (100)	10 (100)	1 (100)	1 (100)	3 (100)
**Streptocyclicin**						
**CC**	**Pre-PCV**	**Post-PCV**	**Carriage**	**IPD**	**LRTI**	**OM**
CC439	107 (100)	110 (100)	127 (100)	17 (100)	22 (100)	51 (100)
CC199	99 (100)	80 (100)	110 (100)	13 (100)	11 (100)	45 (100)
CC97	34 (100)	53 (100)	48 (100)	6 (100)	9 (100)	24 (100)
CC124	44 (100)	18 (100)	29 (100)	12 (100)	7 (100)	14 (100)
CC392	24 (88.9)	19 (95.0)	31 (96.9)	1 (33.3)	5 (100)	6 (85.7)
CC156/162	36 (92.3)	7 (100)	14 (82.4)	11 (100)	10 (100)	8 (100)
CC30	24 (70.6)	19 (73.1)	32 (80.0)	1 (50.0)	6 (60.0)	4 (50.0)
CC1262	6 (100)	29 (100)	21 (100)	2 (100)	7 (100)	5 (100)
CC344	14 (87.5)	21 (100)	31 (93.9)	0	4 (100)	0

CC193	6 (100)	22 (100)	16 (100)	2 (100)	5 (100)	5 (100)
Other CCs	33 (71.7)	55 (53.9)	50 (61.7)	16 (55.2)	11 (50.0)	11 (68.8)
Other Singletons	0	2 (100)	1 (100)	0	0	1 (100)
**Streptolancidin A**						
**CC**	**Pre-PCV**	**Post-PCV**	**Carriage**	**IPD**	**LRTI**	**OM**
CC138/176	79 (100)	43 (100)	87 (100)	5 (100)	12 (100)	18 (100)
CC448	15 (100)	14 (100)	27 (100)	0	2 (100)	0
CC338	1 (20.0)	0	0	1 (50.0)	0	0
**Streptolancidin C**						
**CC**	**Pre-PCV**	**Post-PCV**	**Carriage**	**IPD**	**LRTI**	**OM**
CC236/271/320	206 (100)	87 (100)	53 (100)	6 (100)	77 (100)	157 (100)
CC138/176	79 (100)	43 (100)	87 (100)	5 (100)	12 (100)	18 (100)
CC180	64 (100)	43 (100)	55 (100)	9 (100)	21 (100)	22 (100)
CC439	10 (9.3)	32 (29.1)	31 (24.4)	2 (11.8)	3 (13.6)	6 (11.8)
CC15	32 (100)	4 (100)	14 (100)	9 (100)	5 (100)	8 (100)
CC30	15 (44.1)	19 (73.1)	20 (50.0)	2 (100)	6 (60.0)	6 (75.0)
CC448	15 (100)	14 (100)	27 (100)	0	2 (100)	0
CC90	15 (100)	7 (100)	8 (100)	1 (100)	7 (100)	6 (100)
CC393	16 (100)	4 (100)	16 (100)	2 (100)	0	2 (100)
CC191	11 (100)	5 (100)	0	14 (100)	1 (100)	1 (100)
Other CCs	29 (100)	34 (100)	26 (100)	11 (100)	13 (100)	13 (100)
Other Singletons	3 (100)	11 (100)	10 (100)	0	1 (100)	3 (100)
**Streptolancidin D**						
**CC**	**Pre-PCV**	**Post-PCV**	**Carriage**	**IPD**	**LRTI**	**OM**
CC439	51 (47.7)	30 (27.3)	34 (26.8)	9 (52.9)	10 (45.5)	28 (54.9)
CC15	32 (100)	4 (100)	14 (100)	9 (100)	5 (100)	8 (100)
CC30	5 (14.7)	12 (46.2)	12 (30.0)	1 (50.0)	2 (20.0)	2 (25.0)
CC2755	8 (100)	8 (100)	4 (100)	3 (100)	5 (100)	4 (100)
CC177	6 (100)	8 (100)	5 (100)	0	3 (100)	6 (100)
Sing1801	2 (100)	10 (100)	9 (100)	0	1 (100)	2 (100)
CC473	1 (100)	1 (100)	1 (100)	0	0	1 (100)
CC102	2 (100)	0	2 (100)	0	0	0
CC338	2 (40.0)	0	0	0	1 (50.0)	1 (50.0)
CC1766	1 (100)	0	0	0	1 (100)	0
Other CCs	1 (100)	0	0	0	0	1 (25.0)
Other Singletons	0	0	0	0	0	0
**Streptolancidin E**						
**CC**	**Pre-PCV**	**Post-PCV**	**Carriage**	**IPD**	**LRTI**	**OM**
CC439	107 (100)	110 (100)	127 (100)	17 (100)	22 (100)	51 (100)
CC199	97 (98.0)	77 (96.2)	108 (98.2)	13 (100)	10 (90.9)	43 (95.6)
CC344	16 (100)	21 (100)	33 (100)	0	4 (100)	0
CC448	15 (100)	14 (100)	27 (100)	0	2 (100)	0

CC113	12 (85.7)	6 (85.7)	12 (80.0)	2 (100)	2 (100)	2 (100)
CC66	8 (88.9)	7 (70.0)	9 (90.0)	4 (66.7)	1 (50.0)	1 (100)
CC3017	6 (100)	2 (100)	2 (100)	3 (100)	3 (100)	0
CC432	0	4 (66.7)	3 (60.0)	0	0	1 (100)
CC306	1 (50.0)	2 (66.7)	0	3 (60.0)	0	0
CC230	1 (100)	2 (100)	0	1 (100)	0	2 (100)
Other CCs	7 (17.5)	4 (13.8)	3 (7.3)	3 (60.0)	5 (33.3)	0
Other Singletons	1 (100)	1 (100)	1 (100)	1 (100)	0	0
**Streptolancidin F**						
**CC**	**Pre-PCV**	**Post-PCV**	**Carriage**	**IPD**	**LRTI**	**OM**
CC344	14 (87.5)	19 (90.5)	29 (87.9)	0	4 (100)	0
CC100	15 (100)	10 (100)	10 (100)	7 (100)	4 (100)	4 (100)
CC191	11 (100)	5 (100)	0	14 (100)	1 (100)	1 (100)
CC433	7 (53.8)	2 (4.2)	2 (6.2)	2 (15.4)	5 (45.5)	0
CC717	1 (100)	3 (100)	1 (100)	0	0	3 (100)
CC97	2 (5.9)	1 (1.9)	3 (6.2)	0	0	0
CC113	2 (14.3)	0	2 (13.3)	0	0	0
CC346	0	2 (100)	1 (100)	0	0	1 (100)
Sing10346	0	1 (100)	1 (100)	0	0	0
**Streptolancidin G**						
**CC**	**Pre-PCV**	**Post-PCV**	**Carriage**	**IPD**	**LRTI**	**OM**
CC433	13 (100)	48 (100)	32 (100)	13 (100)	11 (100)	5 (100)
CC392	27 (100)	20 (100)	32 (100)	3 (100)	5 (100)	7 (100)
CC393	16 (100)	4 (100)	16 (100)	2 (100)	0	2 (100)
CC66	9 (100)	9 (90.0)	10 (100)	5 (83.3)	2 (100)	1 (100)
CC30	10 (29.4)	7 (26.9)	8 (20.0)	1 (50.0)	4 (40.0)	4 (50.0)
CC2755	8 (100)	8 (100)	4 (100)	3 (100)	5 (100)	4 (100)
CC315	4 (66.7)	9 (100)	4 (80.0)	1 (100)	2 (100)	6 (85.7)
CC15	12 (37.5)	1 (25.0)	8 (57.1)	0	3 (60.0)	2 (25.0)
CC198	0	13 (100)	11 (100)	0	1 (100)	1 (100)
Sing1801	2 (100)	10 (100)	9 (100)	0	1 (100)	2 (100)
Other CCs	8 (100)	14 (73.7)	7 (63.6)	9 (100)	2 (100)	4 (80.0)
Other Singletons	0	0	0	0	0	0
**Streptolancidin J**						
**CC**	**Pre-PCV**	**Post-PCV**	**Carriage**	**IPD**	**LRTI**	**OM**
CC199	86 (86.9)	67 (83.8)	97 (88.2)	11 (84.6)	7 (63.6)	38 (84.4)
CC138/176	79 (100)	43 (100)	87 (100)	5 (100)	12 (100)	18 (100)
CC180	64 (100)	43 (100)	55 (100)	9 (100)	21 (100)	22 (100)
CC97	34 (100)	52 (98.1)	48 (100)	6 (100)	8 (88.9)	24 (100)
CC490	39 (97.5)	32 (94.1)	44 (95.7)	5 (100)	9 (100)	13 (92.9)
CC124	44 (100)	18 (100)	29 (100)	12 (100)	7 (100)	14 (100)
CC433	13 (100)	48 (100)	32 (100)	13 (100)	11 (100)	5 (100)

CC30	34 (100)	26 (100)	40 (100)	2 (100)	10 (100)	8 (100)
CC392	27 (100)	20 (100)	32 (100)	3 (100)	5 (100)	7 (100)
CC156/162	38 (97.4)	7 (100)	17 (100)	10 (90.9)	10 (100)	8 (100)
Other CCs	66 (60.6)	113 (57.4)	91 (52.3)	24 (68.6)	29 (63.0)	35 (68.6)
Other Singletons	2 (100)	10 (100)	9 (100)	0	1 (100)	2 (100)
**Streptolassin**						
**CC**	**Pre-PCV**	**Post-PCV**	**Carriage**	**IPD**	**LRTI**	**OM**
CC392	27 (100)	20 (100)	32 (100)	3 (100)	5 (100)	7 (100)
CC433	0	1 (2.1)	1 (3.1)	0	0	0

Note: The 10 most common clonal complexes in which the bacteriocin cluster was found are listed separately, and the remainder were pooled as 'Other'. Only bacteriocin clusters with significant differences in prevalence in Figure 2B and 2C are included in this table. IPD, invasive pneumococcal disease, LRTI, lower respiratory tract infection, OM, otitis media.

Supplementary Table 10: Bacteriocin clusters in the Kenyan dataset.

Number of pneumococci harbouring each bacteriocin cluster, stratified by CC<br>n (% of CC representatives in each subset with the bacteriocin)				
**Streptococcin A**				
**CC**	**Pre-PCV**	**Post-PCV**	**Carriage**	**IPD**
CC5902	101 (100)	135 (97.8)	219 (98.6)	17 (100)
CC217	199 (99.5)	23 (100)	16 (100)	206 (99.5)
CC701	67 (93.1)	80 (87.9)	130 (89.7)	17 (94.4)
CC1146	53 (100)	86 (100)	126 (100)	13 (100)
CC5339	106 (97.2)	33 (100)	122 (97.6)	17 (100)
CC156/162	36 (100)	95 (100)	97 (100)	34 (100)
CC138/176	66 (98.5)	64 (97.0)	97 (98.0)	33 (97.1)
CC991	24 (100)	80 (100)	95 (100)	9 (100)
CC852	26 (96.3)	51 (100)	66 (98.5)	11 (100)
CC63	56 (100)	14 (100)	37 (100)	33 (100)
Other CCs	576 (81.5)	472 (82.8)	801 (81.2)	247 (85.2)
Other Singletons	42 (100)	74 (98.7)	100 (99.0)	16 (100)
**Streptococcin D**				
**CC**	**Pre-PCV**	**Post-PCV**	**Carriage**	**IPD**
CC63	56 (100)	14 (100)	37 (100)	33 (100)
CC13215	0	14 (100)	14 (100)	0
Sing14766	1 (100)	0	0	1 (100)
**Streptococcin E**				
**CC**	**Pre-PCV**	**Post-PCV**	**Carriage**	**IPD**
CC5902	101 (100)	138 (100)	222 (100)	17 (100)
CC217	200 (100)	23 (100)	16 (100)	207 (100)
CC701	72 (100)	91 (100)	145 (100)	18 (100)
CC5339	109 (100)	33 (100)	125 (100)	17 (100)
CC1146	53 (100)	86 (100)	126 (100)	13 (100)
CC138/176	67 (100)	66 (100)	99 (100)	34 (100)
CC156/162	36 (100)	95 (100)	97 (100)	34 (100)
CC991	24 (100)	80 (100)	95 (100)	9 (100)
CC230	50 (100)	42 (100)	60 (100)	32 (100)
CC852	27 (100)	51 (100)	67 (100)	11 (100)
Other CCs	857 (98.6)	671 (96.1)	1166 (96.8)	362 (99.7)
Other Singletons	49 (100)	94 (100)	127 (100)	16 (100)
**Streptocyclicin**				
**CC**	**Pre-PCV**	**Post-PCV**	**Carriage**	**IPD**
CC5902	78 (77.2)	89 (64.5)	152 (68.5)	15 (88.2)
CC701	72 (100)	91 (100)	145 (100)	18 (100)
CC156/162	36 (100)	95 (100)	97 (100)	34 (100)
CC991	24 (100)	80 (100)	95 (100)	9 (100)

CC230	48 (96.0)	41 (97.6)	58 (96.7)	31 (96.9)
CC852	27 (100)	51 (100)	67 (100)	11 (100)
CC5258	18 (100)	59 (100)	72 (100)	5 (100)
CC289	64 (100)	5 (100)	3 (100)	66 (100)
CC914	43 (97.7)	17 (100)	44 (97.8)	16 (100)
CC702	18 (100)	40 (100)	56 (100)	2 (100)
Other CCs	213 (45.6)	257 (71.2)	395 (58.1)	75 (50.7)
Other Singletons	23 (100)	34 (100)	51 (100)	6 (100)
**Streptolancidin B**				
**CC**	**Pre-PCV**	**Post-PCV**	**Carriage**	**IPD**
CC702	17 (94.4)	40 (100)	55 (98.2)	2 (100)
CC499	36 (100)	19 (100)	44 (100)	11 (100)
CC5902	16 (15.8)	16 (11.6)	32 (14.4)	0
Sing11162	0	23 (100)	20 (100)	3 (100)
CC347	16 (28.1)	2 (40.0)	12 (25.0)	6 (42.9)
CC5250/5947/15006	10 (100)	8 (100)	16 (100)	2 (100)
CC703	9 (100)	7 (100)	14 (100)	2 (100)
CC385	10 (41.7)	3 (42.9)	6 (37.5)	7 (46.7)
CC1264	3 (100)	8 (100)	11 (100)	0
CC6446/14764	2 (100)	9 (100)	9 (100)	2 (100)
Other CCs	40 (40.0)	22 (27.8)	55 (36.7)	7 (24.1)
Other Singletons	10 (100)	12 (100)	19 (100)	3 (100)
**Streptolancidin C**				
**CC**	**Pre-PCV**	**Post-PCV**	**Carriage**	**IPD**
CC5902	101 (100)	138 (100)	222 (100)	17 (100)
CC217	200 (100)	23 (100)	16 (100)	207 (100)
CC5339	106 (97.2)	32 (97.0)	121 (96.8)	17 (100)
CC138/176	67 (100)	66 (100)	99 (100)	34 (100)
CC156/162	36 (100)	95 (100)	97 (100)	34 (100)
CC852	27 (100)	51 (100)	67 (100)	11 (100)
CC289	64 (100)	5 (100)	3 (100)	66 (100)
CC499	36 (100)	17 (89.5)	42 (95.5)	11 (100)
CC7689	36 (100)	3 (100)	30 (100)	9 (100)
CC338	16 (100)	21 (100)	29 (100)	8 (100)
Other CCs	285 (66.6)	301 (72.7)	485 (70.0)	101 (67.8)
Other Singletons	24 (88.9)	55 (94.8)	70 (92.1)	9 (100)
**Streptolancidin D**				
**CC**	**Pre-PCV**	**Post-PCV**	**Carriage**	**IPD**
CC701	71 (98.6)	90 (98.9)	143 (98.6)	18 (100)
CC5339	107 (98.2)	32 (97.0)	122 (97.6)	17 (100)
CC991	24 (100)	80 (100)	95 (100)	9 (100)
CC5902	45 (44.6)	38 (27.5)	75 (33.8)	8 (47.1)

CC854	52 (100)	5 (100)	38 (100)	19 (100)
CC706	30 (100)	7 (100)	27 (100)	10 (100)
Sing11162	0	23 (100)	20 (100)	3 (100)
CC14774	6 (100)	17 (100)	21 (100)	2 (100)
CC4368	17 (94.4)	5 (100)	17 (94.4)	5 (100)
CC5938	9 (100)	9 (90.0)	16 (94.1)	2 (100)
Other CCs	68 (43.0)	82 (46.6)	131 (51.8)	19 (23.5)
Other Singletons	14 (100)	20 (100)	29 (100)	5 (100)
**Streptolancidin E**				
**CC**	**Pre-PCV**	**Post-PCV**	**Carriage**	**IPD**
CC1146	45 (84.9)	54 (62.8)	87 (69.0)	12 (92.3)
CC230	48 (96.0)	40 (95.2)	57 (95.0)	31 (96.9)
CC5258	18 (100)	58 (98.3)	71 (98.6)	5 (100)
CC1381	41 (100)	8 (100)	31 (100)	18 (100)
CC705/14790	8 (100)	30 (100)	33 (100)	5 (100)
CC138/176	5 (7.5)	17 (25.8)	17 (17.2)	5 (14.7)
CC5349	6 (100)	11 (100)	17 (100)	0
CC14858	5 (100)	11 (73.3)	14 (77.8)	2 (100)
CC14892	1 (16.7)	13 (81.2)	14 (63.6)	0
Sing14868	5 (100)	9 (100)	12 (100)	2 (100)
Other CCs	29 (20.3)	42 (21.2)	55 (18.2)	16 (42.1)
Other Singletons	4 (100)	19 (100)	22 (100)	1 (100)
**Streptolancidin G**				
**CC**	**Pre-PCV**	**Post-PCV**	**Carriage**	**IPD**
CC1146	48 (90.6)	86 (100)	121 (96.0)	13 (100)
CC852	27 (100)	51 (100)	67 (100)	11 (100)
CC5329	14 (93.3)	23 (100)	33 (97.1)	4 (100)
CC393	4 (100)	5 (100)	5 (100)	4 (100)
CC5902	0	3 (2.2)	3 (1.4)	0
CC5796	2 (15.4)	0	2 (14.3)	0
CC14774	0	2 (11.8)	2 (9.5)	0
CC909	1 (100)	1 (100)	2 (100)	0
Sing14823	1 (100)	0	1 (100)	0
CC473	1 (25.0)	0	1 (33.3)	0
Other CCs	1 (50.0)	1 (7.1)	2 (15.4)	0
Other Singletons	0	1 (100)	1 (100)	0
**Streptolassin**				
**CC**	**Pre-PCV**	**Post-PCV**	**Carriage**	**IPD**
CC289	64 (100)	5 (100)	3 (100)	66 (100)
CC13854/15057	0	3 (100)	3 (100)	0
CC404	1 (100)	0	1 (100)	0
CC5936/14865	1 (100)	0	1 (100)	0

Sing5359	1 (100)	0	1 (100)	0
Sing14840	1 (100)	0	1 (100)	0
CC5068	1 (33.3)	0	0	1 (50.0)

Note: The 10 most common clonal complexes in which the bacteriocin cluster was found are listed separately, and the remainder were pooled as ‘Other’. Only bacteriocin clusters with significant differences in prevalence in Figure 2B and 2C are included in this table. IPD, invasive pneumococcal disease.

Supplementary Table 11: Clonal complexes (CCs) and sequence types (STs) in the Icelandic dataset with multiple bacteriocin repertoires.

CC	Variable bacteriocins (CC)	Mixed STs	Variable bacteriocins (ST)
236/271/320	Streptococcin A, Streptococcin E	271	Streptococcin E
		1968	Streptococcin A
439	Streptococcin A, Streptolancidin C, Streptolancidin D	311	Streptococcin A
		507	Streptococcin A
		442	Streptococcin A
		190	Streptococcin A
199	Streptolancidin J, Streptosactin	199	Streptolancidin J, Streptosactin
138/176	Streptococcin A	176	Streptococcin A
180	Streptococcin A	180	Streptococcin A
62	Streptococcin A, Streptolancidin J	62	Streptolancidin J
97	Streptococcin A, Streptolancidin F, Streptolancidin J	1635	Streptolancidin J
490	Streptolancidin J	2221	Streptolancidin J
124	Streptococcin A	124	Streptococcin A
433	Streptocyclicin, Streptolancidin F, Streptolassin	433	Streptolancidin F
30	Streptocyclicin, Streptolancidin C, Streptolancidin D, Streptolancidin E, Streptolancidin G	30	Streptolancidin E
392	Streptocyclicin	440	Streptocyclicin
156/162	Streptocyclicin, Streptolancidin J	162	Streptolancidin J
344	Streptocyclicin, Streptolancidin F, Streptolancidin K	10371	Streptocyclicin, Streptolancidin F
		344	Streptolancidin F, Streptolancidin K
15	Streptolancidin G	None	NA
1262	Streptolancidin J	1262	Streptolancidin J
193	Streptolancidin J	1877	Streptolancidin J
113	Streptococcin A, Streptolancidin F	113	Streptococcin A, Streptolancidin F
113	Streptococcin A, Streptolancidin F	110	Streptococcin A
393	Streptococcin A	None	NA
66	Streptolancidin G	None	NA

315	Streptolancidin G, Streptolancidin J	386	Streptolancidin J
6524	Streptolancidin J	6524	Streptolancidin J
338	Streptolancidin A, Streptolancidin D	None	NA
63	Streptolancidin D	None	NA
432	Streptolancidin G	432	Streptolancidin G
205	Streptolancidin J	205	Streptolancidin J
306	Streptococcin A	None	NA
230	Streptolancidin J	None	NA
473	Streptolancidin G	None	NA

Supplementary Table 12: Clonal complexes (CCs) and sequence types (STs) in the Kenyan dataset with multiple bacteriocin repertoires.

CC	Variable bacteriocins (CC)	Mixed STs	Variable bacteriocins (ST)
5902	Streptococcin A, Streptocyclicin, Streptolancidin B, Streptolancidin D, Streptolancidin E, Streptolancidin G, Streptolancidin J	5902	Streptocyclicin, Streptolancidin J
		5370	Streptococcin A, Streptolancidin E
		840	Streptolancidin D
		2052	Streptolancidin E
		15056	Streptolancidin G
217	Streptococcin A, Streptolancidin E	613	Streptococcin A, Streptolancidin E
701	Streptococcin A, Streptolancidin D, Streptolancidin J	701	Streptococcin A, Streptolancidin D
		5340	Streptococcin A
5339	Streptococcin A, Streptocyclicin, Streptolancidin C, Streptolancidin D, Streptolancidin J	5339	Streptococcin A, Streptolancidin D
		844	Streptococcin A, Streptocyclicin, Streptolancidin C, Streptolancidin D
		5367	Streptolancidin J
		5268	Streptococcin A, Streptolancidin C
1146	Streptolancidin E, Streptolancidin G, Streptolancidin J	5952	Streptolancidin E
		5396	Streptolancidin G
138/176	Streptococcin A, Streptocyclicin, Streptolancidin A, Streptolancidin E, Streptolancidin J	848	Streptococcin A, Streptolancidin E, Streptolancidin J
156/162	Streptolancidin E, Streptolancidin K	847	Streptolancidin E, Streptolancidin K
230	Streptocyclicin, Streptolancidin D, Streptolancidin E, Streptolancidin F, Streptolancidin J	230	Streptolancidin D, Streptolancidin J
		700	Streptocyclicin
		4351	Streptolancidin E
852	Streptococcin A	852	Streptococcin A
5258	Streptococcin A	5258	Streptococcin A
63	Streptolancidin C, Streptolancidin J	842	Streptolancidin J
		2716	Streptolancidin C

347	Streptococcin A, Streptocyclicin, Streptolancidin B, Streptolancidin C, Streptolancidin J	6088	Streptococcin A, Streptolancidin J
		2715	Streptolancidin B, Streptolancidin J
		5769	Streptococcin A, Streptolancidin J
		6095	Streptolancidin J
		14817	Streptococcin A, Streptolancidin J
914	Streptolancidin B	None	NA
7053	Streptococcin A, Streptolancidin C, Streptolancidin J	5368	Streptolancidin J
702	Streptolancidin B, Streptolancidin C	702	Streptolancidin B, Streptolancidin C
854	Streptococcin A, Streptocyclicin, Streptolancidin J	854	Streptococcin A, Streptocyclicin, Streptolancidin J
499	Streptolancidin C, Streptolancidin J	499	Streptolancidin C
		5907	Streptolancidin J
5329	Streptocyclicin, Streptolancidin C, Streptolancidin G, Streptolancidin J, Streptolancidin K	5329	Streptolancidin C, Streptolancidin J
338	Streptolancidin D, Streptolancidin E, Streptolancidin J	172	Streptolancidin E
		2054	Streptolancidin D, Streptolancidin J
3460	Streptococcin A, Streptocyclicin, Streptolancidin B	14886	Streptococcin A, Streptocyclicin
		3460	Streptococcin A
385	Streptococcin A, Streptolancidin B, Streptolancidin J	6097	Streptolancidin B
		2713	Streptococcin A
		3207	Streptolancidin J
989	Streptolancidin D	989	Streptolancidin D
14774	Streptolancidin B, Streptolancidin G, Streptolancidin J	6092	Streptolancidin B, Streptolancidin G
		14774	Streptolancidin J
14892	Streptococcin A, Streptolancidin C, Streptolancidin D, Streptolancidin E	None	NA

2386	Streptolancidin C, Streptolancidin D	5331	Streptolancidin D
14858	Streptocyclicin, Streptolancidin E	14858	Streptolancidin E
		14910	Streptocyclicin
4894	Streptococcin A	4894	Streptococcin A
5938	Streptolancidin D	None	NA
14930/15024	Streptocyclicin, Streptolancidin C, Streptolancidin J	14930	Streptocyclicin, Streptolancidin C, Streptolancidin J
5349	Streptococcin A	None	NA
5294	Streptococcin A, Streptocyclicin	5294	Streptococcin A, Streptocyclicin
703	Streptocyclicin, Streptolancidin C, Streptolancidin J	703	Streptolancidin C, Streptolancidin J
Sing5373	Streptolancidin C	5373	Streptolancidin C
5372/15025	Streptolancidin G	5372	Streptolancidin G
Sing14868	Streptococcin A	14868	Streptococcin A
5796	Streptococcin A, Streptolancidin G, Streptolancidin J	5796	Streptococcin A
5560/6090/6103	Streptococcin A	6103	Streptococcin A
193	Streptolancidin C, Streptolancidin J	None	NA
1766	Streptococcin A	None	NA
1264	Streptolancidin D	1264	Streptolancidin D
3735	Streptocyclicin	None	NA
14846/14876	Streptococcin A, Streptolancidin D	14846	Streptococcin A, Streptolancidin D
5798/5879	Streptococcin A, Streptolancidin B	5798	Streptococcin A, Streptolancidin B
5321/14966	Streptolancidin C	None	NA
393	Streptolancidin J	None	NA
547	Streptolancidin D, Streptolancidin J	None	NA
3518	Streptococcin A, Streptolancidin B	None	NA
3983	Streptococcin A	3983	Streptococcin A
5266	Streptolancidin J	None	NA
5901	Streptococcin A	14976	Streptococcin A
5398/14814	Streptolancidin C	None	NA
473	Streptolancidin G	None	NA

5839/14990	Streptolancidin J	None	NA
5068	Streptolancidin J, Streptolassin	None	NA
Sing5376	Streptolancidin J	5376	Streptolancidin J
849/5343/5351	Streptococcin A	None	NA

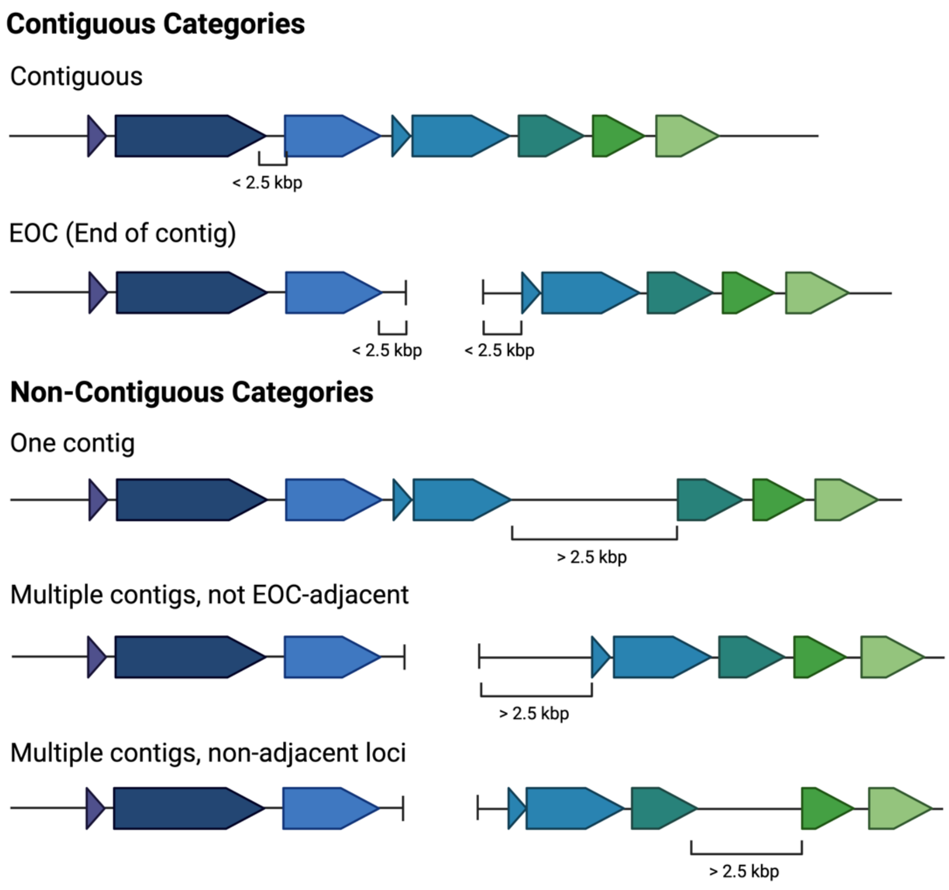

Supplementary Figure 1: Illustration of a hypothetical bacteriocin biosynthetic gene cluster and the various cluster contiguity categories quantified in Supplementary Table 6 (figure created using BioRender).
